# Oxidative Deamination of Serum Albumins by (-)-Epigallocatechin-3-*O*-Gallate: A Potential Mechanism for the Formation of Innate Antigens by Antioxidants

**DOI:** 10.1371/journal.pone.0153002

**Published:** 2016-04-05

**Authors:** Yukinori Hatasa, Miho Chikazawa, Mai Furuhashi, Fumie Nakashima, Takahiro Shibata, Tatsuhiko Kondo, Mitsugu Akagawa, Hiroki Hamagami, Hiroshi Tanaka, Hirofumi Tachibana, Koji Uchida

**Affiliations:** 1 Graduate School of Bioagricultural Sciences, Nagoya University, Nagoya, Japan; 2 PRESTO, Japan Science and Technology Agency, Saitama, Japan; 3 Department of Biological Chemistry, Division of Applied Life Science, Graduate School of Life and Environmental Sciences, Osaka Prefecture University, Sakai, Japan; 4 Graduate School of Science and Engineering, Tokyo Institute of Technology, Tokyo, Japan; 5 Faculty of Agriculture, Kyushu University, Fukuoka, Japan; Institute of Nutrition, GERMANY

## Abstract

(-)-Epigallocatechin-3-*O*-gallate (EGCG), the most abundant polyphenol in green tea, mediates the oxidative modification of proteins, generating protein carbonyls. However, the underlying molecular mechanism remains unclear. Here we analyzed the EGCG-derived intermediates generated upon incubation with the human serum albumin (HSA) and established that EGCG selectively oxidized the lysine residues via its oxidative deamination activity. In addition, we characterized the EGCG-oxidized proteins and discovered that the EGCG could be an endogenous source of the electrically-transformed proteins that could be recognized by the natural antibodies. When HSA was incubated with EGCG in the phosphate-buffered saline (pH 7.4) at 37°C, the protein carbonylation was associated with the formation of EGCG-derived products, such as the protein-bound EGCG, oxidized EGCG, and aminated EGCG. The aminated EGCG was also detected in the sera from the mice treated with EGCG *in vivo*. EGCG selectively oxidized lysine residues at the EGCG-binding domains in HSA to generate an oxidatively deaminated product, aminoadipic semialdehyde. In addition, EGCG treatment results in the increased negative charge of the protein due to the oxidative deamination of the lysine residues. More strikingly, the formation of protein carbonyls by EGCG markedly increased its cross-reactivity with the natural IgM antibodies. These findings suggest that many of the beneficial effects of EGCG may be partly attributed to its oxidative deamination activity, generating the oxidized proteins as a target of natural antibodies.

## Introduction

Oxidative modification of proteins and subsequent accumulation of oxidized proteins, an indication of oxidative tissue damage, has been reported in diseased tissues from age-related pathologies, such as atherosclerosis, neurodegenerative disorders, and cataract [1]. There is ample evidence to support the notion that the most important mechanism of oxidative damage to proteins is metal-catalyzed oxidation. This process includes the generation of H_2_O_2_ and reduction of Fe^3+^ or Cu^2+^ by a suitable electron donor; Fe^2+^ and Cu^+^ ions bind to specific metal binding sites on proteins and react with H_2_O_2_ to generate reactive oxygen species, such as the hydroxyl radical. They can lead to oxidation of amino acid residue side chains, formation of protein-protein cross-linkages, and oxidation of the protein backbone resulting in protein fragmentation. It has been shown that some of the oxidized amino acid residues have a carbonyl functionality [2]. It has been established that the protein carbonyls accumulate on tissue proteins during aging and disease development [3, 4]. Although the experimental evidence is so far mostly correlative, it lends strong support to the hypothesis that the protein carbonyl content of tissues reflects the fraction of oxidatively damaged protein with impaired function and might therefore be at the root of disease and aging-related functional losses. It is generally considered that these oxidized proteins are degraded by the proteasome. The oxidized proteins could also be incompletely degraded and accumulate within the lysosomal compartments resulting in the formation of lipofuscin-like, autofluorescent aggregates [5]. It is therefore likely that these oxidized proteins might constitute a previously unrecognized but important class of damage-associated molecular patterns. However, biological function of oxidized proteins, especially whether they could function as damage-associated molecular patterns, remains poorly understood. Here, we show that oxidative modification of proteins, including oxidative deamination of lysine residues in proteins, could lead to the formation of innate epitopes. Given the large number of studies suggesting the involvement of oxidative protein modification in diseases, the significance of the findings in this report may be far-reaching.

Diets rich in fruits and vegetables are associated with a reduced risk of several major diseases, including cancers, diabetes, hypertension, and heart disease [6. 7]. It has been shown that non-nutritive plant chemicals called phytochemicals in fruits and vegetables play a critical role in these beneficial effects. Polyphenols, among the phytochemicals, have received much attention in disease prevention due to their unique chemical and biological properties. Polyphenols are antioxidants in general. However, they also activate adaptive cellular response pathways in a variety of cells and modulate both the innate and adaptive immune responses. Dietary small molecules are absorbed through the lining of the small intestine into bloodstream where they bind to serum albumins, the most abundant protein in plasma. Serum albumins act as a transporter of an exceptionally broad spectrum of small molecules, whereas they also undergo covalent modification by electrophilic intermediates. Importantly, the modified serum albumins with an oxidized fatty acid metabolite have been shown to act as a ligand called damage-associated molecular patterns in innate immunity, mediating an inflammatory response [8]. More recently, prominent IgM titers to the modified serum albumin with dehydroascorbic acid (DHA), in addition to the native DNA and oxidized LDL, have been detected in the mouse sera [9]. Therefore, it can be speculated that the serum albumins may gain a new function through covalent interaction with small dietary molecules, thereby contributing to biological events associated with the innate immune and inflammatory processes.

(-)-Epigallocatechin-3-*O*-gallate (EGCG) is the most abundant and powerful antioxidant in green tea. EGCG has a very unique potential of mediating the oxidative modification of proteins, generating protein carbonyls [10–12]. It has been speculated that EGCG is oxidized to generate an *O*-quinones derivative (oxEGCG), which covalently binds primary amines (R-CH_2_-NH_2_) to form Schiff-base intermediates, followed by the conversion to the aminated EGCG (NH_2_-EGCG) and deaminated products (R-CHO) [12, 13]. However, to date, no attempt has been made to establish the oxidative deamination activity of EGCG by detecting these EGCG-derived products. In the present study, we analyzed the EGCG-derived intermediates and oxidized amino acids generated in the EGCG-treated human serum albumin (HSA) and established the molecular mechanism of oxidative deamination by EGCG. In addition, we characterized the EGCG-oxidized proteins and found that the EGCG-mediated oxidative modification of the protein was associated with the formation of electronegative molecules that could be recognized by the natural antibodies.

## Materials and Methods

### Materials

Catechins, DHA, *p*-aminobenzoic acid (ABA), and human serum albumin (HSA) (fatty acid and globulin free, purity 99%) were obtained from Sigma-Aldrich. *O*-Methyl derivatives of EGCG were prepared as previously reported [14]. Bovine serum albumin (BSA) and pyrroloquinoline quinone (PQQ) were obtained from Wako Pure Chemicals (Japan). The HRP-linked anti-mouse IgM (for ELISA analysis) was obtained from Cappel Laboratories. Hydrogen peroxide (31%, W/V) was obtained from Mitsubishi Gas Co., Ltd. Horseradish peroxidase (HRP)-conjugated NeutrAvidin and enhanced chemiluminescence (ECL) Western blotting detection reagents were obtained from GE Healthcare. All of the other reagents used in the study were of analytical grade and obtained from commercial sources.

### *In Vitro* Incubation of Serum Albumins

HSA or BSA (1.0 mg/ml) was incubated with 1.0 mM phytochemicals in PBS (pH 7.4) at 37°C under atmospheric oxygen. The metal-catalyzed oxidation of proteins was performed by incubating BSA (1 mg/ml) with 1 mM H_2_O_2_, or 200 μM PQQ in the presence of 100 μM Cu^2+^ in PBS (pH 7.4) at 37°C under atmospheric oxygen.

### Zeta Potential

The zeta potential measurement was performed using a zeta potential analyzer (Zetasizer Nano ZS, Malvern).

### ELISA (Enzyme-Linked Immunosorbent Assay)

The native and modified proteins were used as the antigens. A 100-μl aliquot of the antigen solution (50 μg/ml) was added to each well of a 96-well ELSIA plate (Nunc MaxiSorp) and incubated for overnight at 4°C. The antigen solution was then removed, and the plate was washed three times with PBS containing 0.5% Tween 20 (PBS/Tween). Each well was incubated with 200 μl of 4% Blockace (Yukijirushi, Sapporo, Japan) in PBS/Tween for 60 min at 37°C to block the unsaturated plastic surface. The plate was then washed three times with PBS/Tween. A 100-μl aliquot of a 300~500× dilution of mouse serum or mouse monoclonal IgMs was added to each well and incubated for 2 h at 37°C. After discarding the supernatants and washing three times with PBS/Tween, 100 μl of a 5000× dilution of goat anti-mouse IgM conjugated to horseradish peroxidase in PBS/Tween was added. After incubation for 1 h at 37°C, the supernatant was discarded, and the plates were washed three times with PBS/Tween. The enzyme-linked Ab bound to the well was revealed by adding 100 μl/well of 1,2-phenylenediamine (0.5 mg/ml) in a 0.1 M citrate/phosphate buffer (pH 5.5) containing 0.003% hydrogen peroxide. The reaction was terminated by the addition of 2 M sulfuric acid (50 μl/well), and the absorbance at 492 nm was read using a micro-ELISA plate reader. The signals were within the dynamic range of the assays with respect to Ab levels.

### Sulfhydryl Labeling with Maleimide PEG2-Biotin

Aliquots (100 μl) of the protein samples were treated with 0.5 μl of maleimide PEG2-biotin (1 mM) and incubated for 2 h at 4°C. The protein samples were boiled in the Laemmli sample buffer for 5 min, and the biotinylated proteins were then subjected to SDS-PAGE/Western blot followed by detection with HRP-conjugated NeutrAvidin and ECL.

### Detection of Protein Carbonyls

Biotin labeling of the protein carbonyls was performed as previously described [15]. Protein-bound carbonyls were labeled with biotin-LC-hydrazide prior to the treatment with the sample buffer. The protein samples were boiled in the Laemmli sample buffer for 5 min, and the biotinylated proteins were then subjected to SDS-PAGE/Western blot followed by detection with HRP-conjugated NeutrAvidin and ECL.

### Click Chemistry

EGCG-N_3_ for the click chemistry was synthesized from EGCG and 6-azide-6-deoxyl-idose. The manuscript involving the detail of it's synthetic procedure is in preparation (Tanaka, H. et al., submitted for publication). HSA (1.0 mg/ml) was incubated with 1 mM EGCG-N_3_ in PBS (pH 7.4) at 37°C. Click chemistry was performed using the reaction mixtures containing 1.0 mg/ml protein with 1 mM CuSO_4_, 1 mM ascorbic acid, 0.1 mM tris((1-benzyl-1H-1,2,3- triazol-4-yl)methyl)amine (Anaspec, Inc., San Jose), and 20 μM alkyne-PEG4-biotin (Click Chemistry Tools). After incubation in the dark for 2 h at room temperature, the protein samples were boiled in the Laemmli sample buffer for 5 min, and the biotinylated proteins were then subjected to SDS-PAGE/Western blot followed by detection with HRP-conjugated NeutrAvidin and ECL.

### LC-ESI-MS and MS/MS Analysis of Oxidized and Aminated EGCG Derivatives

The conversion of EGCG to the oxidized and aminated derivatives was traced using an ACQUITY TQD system (Waters) equipped with an ESI source in the positive ion mode. The sample injection volumes of 10 μl each were separated on a Develosil HB C30-UG-3 (100 mm × 2.0 mm, Nomura Chemical, Japan) at the flow rate of 0.3 ml/min. A discontinuous gradient was used by solvent A (H_2_O containing 0.1% formic acid) with solvent B (acetonitrile containing 0.1% formic acid) as follows: 1% B at 0 min, 1% B at 1min, 99% B at 8 min. The mass spectrometer was operated in the Selected Ion Recording (SIR) or MRM mode with positive electrospray ionization (ESI+) to analyze the EGCG and its derivatives. The oxidized and aminated EGCGs were quantified as EGCG equivalents. The monitored MRM transitions were as follows: EGCG, m/z 459→139; NH_2_-EGCG, m/z 458→139.

### LC-ESI-MS/MS Analysis of Oxidized Amino Acids

The authentic ABA derivatives of aminoadipic semialdehyde (AAS) and γ-glutamic semialdehyde (GGS) were prepared as previously reported [16]. The protein samples (500 μl) were reductively aminated with 25 mM ABA in 1.1 M HCl and 16 mM NaCNBH_3_ in H_2_O at 37°C in the dark. After reacting for 2 h, the mixture was treated with 500 μl of cold 20% (w/v) trichloroacetic acid (TCA) in an ice bath. After 1h, the mixture was centrifuged at 6,000×g for 30 min, and resulting pellet of precipitated protein was separated. The pellet was washed with 500 μl of 10% (w/v) TCA and 500 μl of cold diethyl ether. The resulting protein was then hydrolyzed for 24 h at 110°C with 6 M HCl. The hydrolysate was dried by centrifugal evaporation *in vacuo* followed by reconstitution in 200 μl of H_2_O. Mass spectrometric analyses were performed using an ACQUITY TQD system (Waters) equipped with an ESI probe and interfaced with a UPLC system (Waters). The sample injection volumes of 10 μl each were separated on a Develosil HB C30-UG-3 (100 mm × 2.0 mm, Nomura Chemical, Japan) at the flow rate of 0.3 ml/min. A discontinuous gradient was used by solvent A (H_2_O containing 0.1% formic acid) with solvent B (acetonitrile containing 0.1% formic acid) as follows: 1% B at 0 min, 1% B at 1min, 99% B at 8 min. Mass spectrometric analyses were performed on line using ESI-MS/MS in the positive ion mode along with the MRM mode (20 eV cone potential /15 eV collision energy). The monitored MRM transitions were as follows: ABA-AAS, m/z 267→84; ABA-GGS, m/z 253→70.

### Identification of AAS Formation Sites in EGCG-Treated Proteins

The EGCG-treated protein was prepared by incubating HSA (1.0 mg/ml) with 1 mM EGCG in PBS at 37°C for 24 h. The protein was proteolyzed with sequence grade modified trypsin (Promega) in 50 mm NH_4_HCO_3_ buffer in the presence of 0.01% Protease MAX surfactant (Promega) for 3 h at 37°C. The resulting peptides were then resolved by reverse-phase nano-LC (DiNa Nano LC system, KYA TECH Corp., Tokyo, Japan) and then directly fractionated onto a MALDI target plate with α-cyano-4-hydroxycinnamic acid by a spotter (DiNa Map system). Mass spectrometry was performed using a MALDI-TOF/TOF MS (AB Sciex TOF/TOF 5800). The MS/MS data were analyzed by MASCOT.

### Molecular Modeling

The AMBER 7.0 force field [17] was used to simulate the effect of the AAS formation on HSA. The crystal structure of the native HSA (Protein Data Bank identification number 1ao6) at 2.5 Å [18] was obtained from the Research Collaboratory for Structural Bioinformatics database. Native and oxidized HSA structures were viewed and manipulated using Molecular Operating Environment (MOE) software (Chemical Computing Group, Montreal, Quebec, Canada). For the molecular dynamics simulation, the hydrogen positions and ionization states were assigned using Protonate 3D application [19]. The protein structures were equilibrated using the Generalized Born solvation model [20] and heated from 0 to 300 K in 100 ps, then subjected to a 2-ns simulation at 300 K. Docking studies were performed using Site Finder and Dock applications in MOE software. The EGCG conformation database produced by the conformational search (38 entries) was used for the query of the docking experiment. The ranking of the generated solutions was performed using the estimated free binding energy, ΔG, of the protein-ligand complex.

### Animals

Female BALB/C, female MRL-MpJ, and female MRL-*lpr* mice were purchased from the Japan SLC (Hamamatsu, Japan). The mice were housed in a temperature-controlled pathogen-free room with light from 7:00 to 19:00 h (daytime) and had free access to standard food and water. All animal works were carried out in accordance with the law (number 105) and notification (number 6) of the Japanese government for the welfare of experimental animal. All procedures were approved by the Animal Experiment Committee in the Graduate School of Bioagricultural Sciences, Nagoya University (Permit Number: 2015030216 and 2015051301). Any animals did not become sick or died prior to the experimental endpoint. After experiments, mice were euthanized by cervical spine fracture dislocation. For *in vivo* experiments, fifteen female BALB/C mice were divided into three groups (n = 5) matched for body weight: vehicle, 10-min treatment, and 30-min treatment. The BALB/c mice (6-weeks old) were intraperitoneally injected with 0.1 ml of EGCG (10 mM) or vehicle control (PBS). After injection for 10 or 30 min, blood was collected by tail bleeds and allowed to stand for 2 h at room temperature, after which the sera were collected by centrifugation at 3,500 rpm for 10 min. The resulting sera (100 μl) were mixed with 500 μl of cold acetone, and incubated for 2 h at -20°C. After centrifugation at 10,000 rpm for 5 min, resulting supernatants were evaporated, redissolved in 200 μl of H_2_O, and then analyzed by LC-ESI-MS/MS. For ELISA analysis, three MRL-MpJ and three MRL-*lpr* mice were used as sera donors at 10–12 weeks old. Blood was collected from tail vein and allowed to stand for 2 h at room temperature, after which the sera were collected by centrifugation at 3,500 rpm for 10 min and stored at -20°C until use.

## Results

### Detection of Protein-Bound EGCG by Click Chemistry

**Fig 1:**
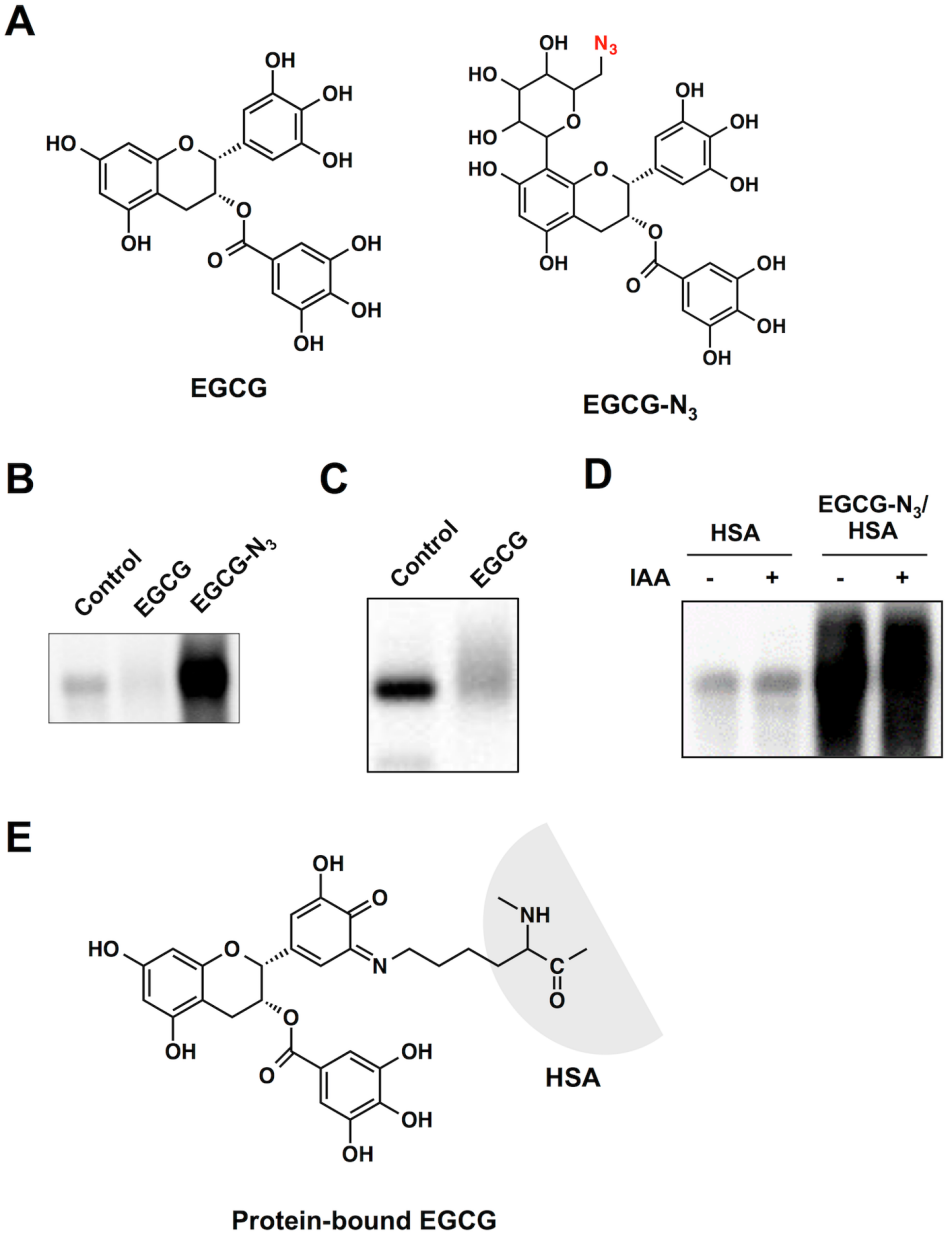
Analysis of a protein-bound EGCG using click chemistry. (**A**) Chemical structures of EGCG and an azide-containing probe (EGCG-N_3_). (**B**) Fluorescent detection of protein-bound EGCG. HSA was pretreated with EGCG-N_3_ or vehicle for 24 h. The reaction mixtures were subjected to the click reaction with an alkynylated biotin followed by separation on SDS-PAGE gel. (**C**) Sulfhydryl modification of HSA by EGCG. NeutrAvidin blot analysis using iodoacetyl-LC-biotin. HSA (1 mg/ml) was incubated with EGCG (1 mM) in 0.1 ml of PBS (pH 7.4) for 24 h at 37°C. (**D**) Effect of IAA pre-treatment on the binding of EGCG-N_3_ to HSA. Native and IAA-treated HSA were incubated with and without EGCG-N_3_ and then subjected to the click chemistry. (**E**) A possible structure of an EGCG-lysine adduct in proteins.

To establish the oxidative deamination activity of EGCG, we sought to detect the EGCG-derived products generated upon incubation of HSA with EGCG. First, to detect the protein-bound EGCG, a probe (EGCG-N_3_) was designed, combining an EGCG-reactive group and an azide functionality (Fig 1A). After exposure of HSA to the EGCG probe, click chemistry was performed with the modified proteins followed by the separation of the proteins on SDS-PAGE. In accordance with the protein carbonyl formation, a dramatic increase in the protein-bound label was observed when the protein was incubated with EGCG-N_3_ (Fig 1B). On the other hand, EGCG is known to form covalent adducts with cysteinyl thiol residues in proteins [10]. Sulfhydryl modification by EGCG was indeed confirmed (Fig 1C). However, the pretreatment of HSA with iodoacetamide (IAA) showed no discernible effect on the click chemistry-mediated binding of EGCG-N_3_ to the protein (Fig 1D). Thus, it appears that the cysteine residue may not be involved in the covalent binding of EGCG to the protein. These data suggest the formation of a lysine-bound EGCG as an intermediate (Fig 1E).

### LC-ESI-MS Analysis of the Oxidized and Aminated EGCGs

**Fig 2:**
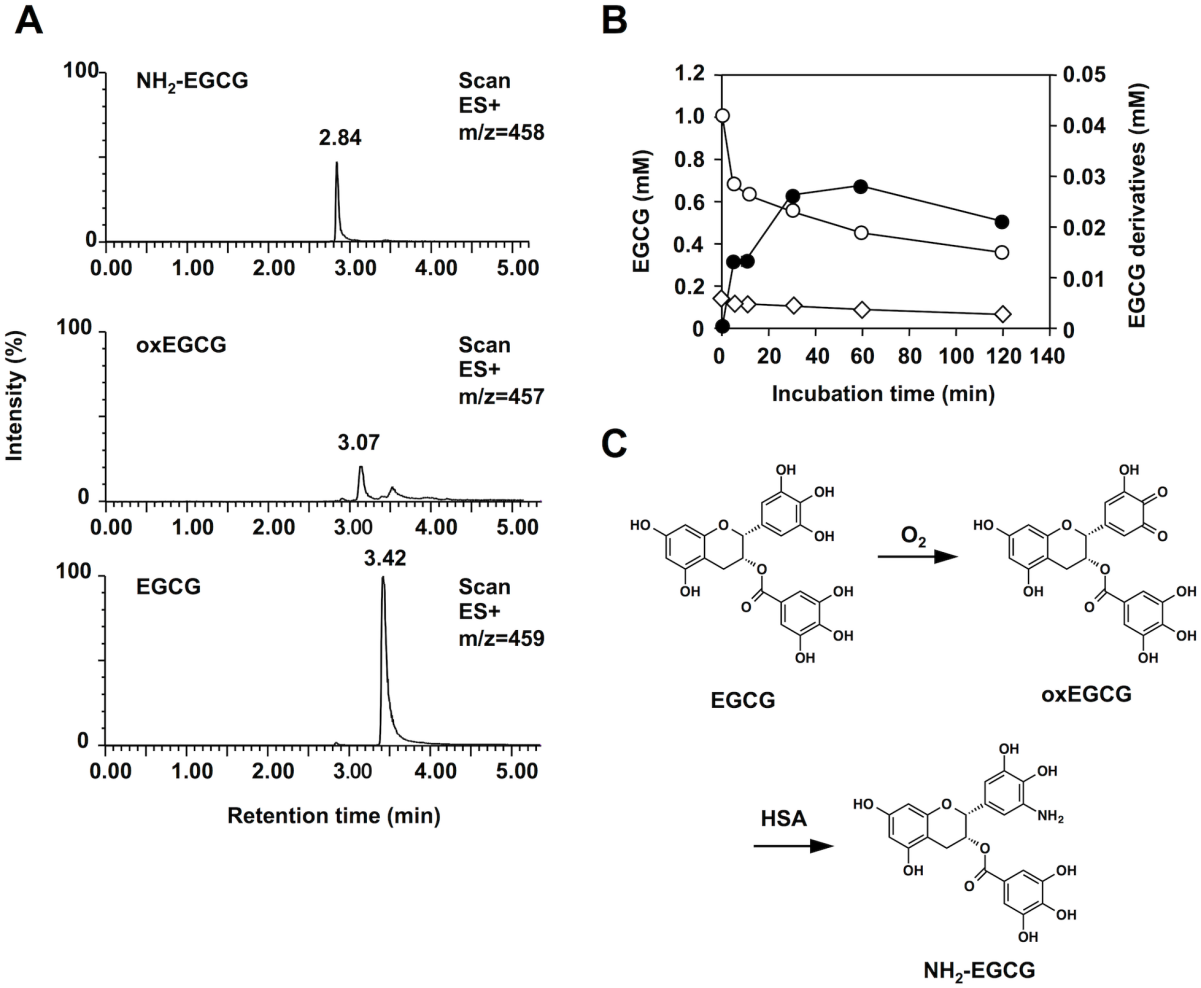
LC-ESI-MS analysis of oxidized and aminated EGCGs. (**A**) Selected ion-current chromatograms monitored with *m/z* 459 (M+H)^+^, *m/z* 457 (M+H)^+^, and *m/z* 458 (M+H)^+^ for EGCG, oxEGCG, and NH_2_-EGCG, respectively. HSA (1 mg/ml) was incubated with 1 mM EGCG in PBS (pH 7.4) at 37°C and the products were analyzed by LC-ESI-MS in the positive ion mode. (**B**) Time-dependent consumption of EGCG and concomitant formation of oxEGCG and NH_2_-EGCG. The EGCG-derived products were quantitated as EGCG equivalents. *Symbols*: *open circle*, EGCG; *open triangle*, oxEGCG; *closed circle*, NH_2_-EGCG. (**C**) A proposed mechanism for the oxidative deamination of lysine residues in proteins by EGCG.

Next, we attempted to detect the EGCG-derived products, oxEGCG and NH_2_-EGCG, generated during the EGCG-mediated oxidation of HSA using LC-ESI-MS. As shown in Fig 2A, when monitored with *m/z* 458 (M+H)^+^ corresponding to the molecular mass of NH_2_-EGCG, the reaction of HSA with EGCG gave a single product. The LC-ESI/MS/MS analysis of the product showed the collision-induced dissociation on *m/z* 458 giving rise to peaks at *m/z* 288 and *m/z* 139 (S1 Fig). Thus, the most likely structure of this product was an aminated EGCG. In addition, the incubation of EGCG with ammonia gave the same product (S2 Fig). However, when monitored with *m/z* 457 (M+H)^+^ corresponding to the molecular mass of oxEGCG, only a trace amount of multiple products was detected. This may be due to the presence of multiple sites in EGCG that could undergo oxidation. The kinetics of the EGCG oxidation and the formation of the oxidized and aminated products showed that, when HSA was incubated with 1 mM EGCG, about 56% of the EGCG was consumed (Fig 2B). The products, corresponding to oxEGCG, were detected even at the initial time point and were gradually consumed with time. The corresponding NH_2_-EGCG was formed in parallel with the loss of EGCG. The amounts corresponded to about 0.5% of EGCG that disappeared during the auto-oxidation. These data provided direct experimental support for the hypothesis that EGCG is oxidized to oxEGCG followed by the conversion to NH_2_-EGCG upon reaction with the protein (Fig 2C).

**Fig 3:**
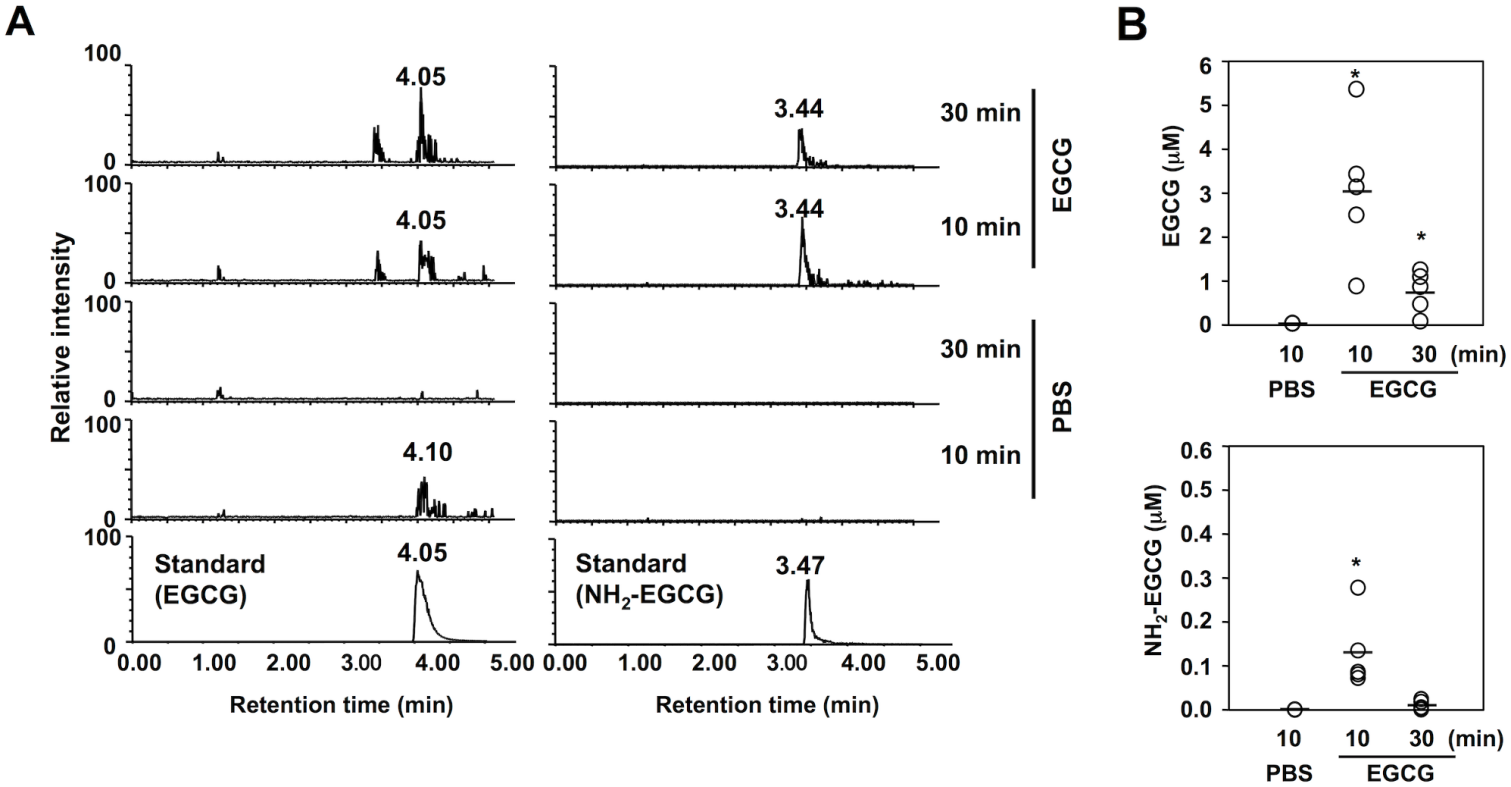
LC-ESI-MS/MS analysis of EGCG and NH_2_-EGCG in the sera of mice treated with EGCG. (**A**) The ion current tracings of EGCG (*left five tracings*) and NH_2_-EGCG (*right five tracings*). BALB/c mice were intraperitoneally injected with 0.1 ml of EGCG (10 mM) or PBS. After injection for 10 or 30 min, the sera were collected. After removing proteins by precipitation with cold acetone, the samples were analyzed by LC-ESI-MS/MS with SRM mode. (**B**) Quantitative analysis of EGCG (*upper panel*) and NH_2_-EGCG (*lower panel*) in the sera of the mice treated with EGCG.

Furthermore, we attempted to detect NH_2_-EGCG *in vivo*. The sera from BALB/C mice intraperitoneally treated with EGCG were analyzed for EGCG and the intermediate using LC-ESI-MS/MS with SRM mode (Fig 3A). The product ion spectra showed the presence of EGCG and NH_2_-EGCG in the sera. The average amount of EGCG and NH_2_-EGCG in the sera after a 10-min treatment with EGCG was 3.1 and 0.13 μM, respectively (Fig 3B).

### Transformation of Lysine Residues into AAS by EGCG

**Fig 4:**
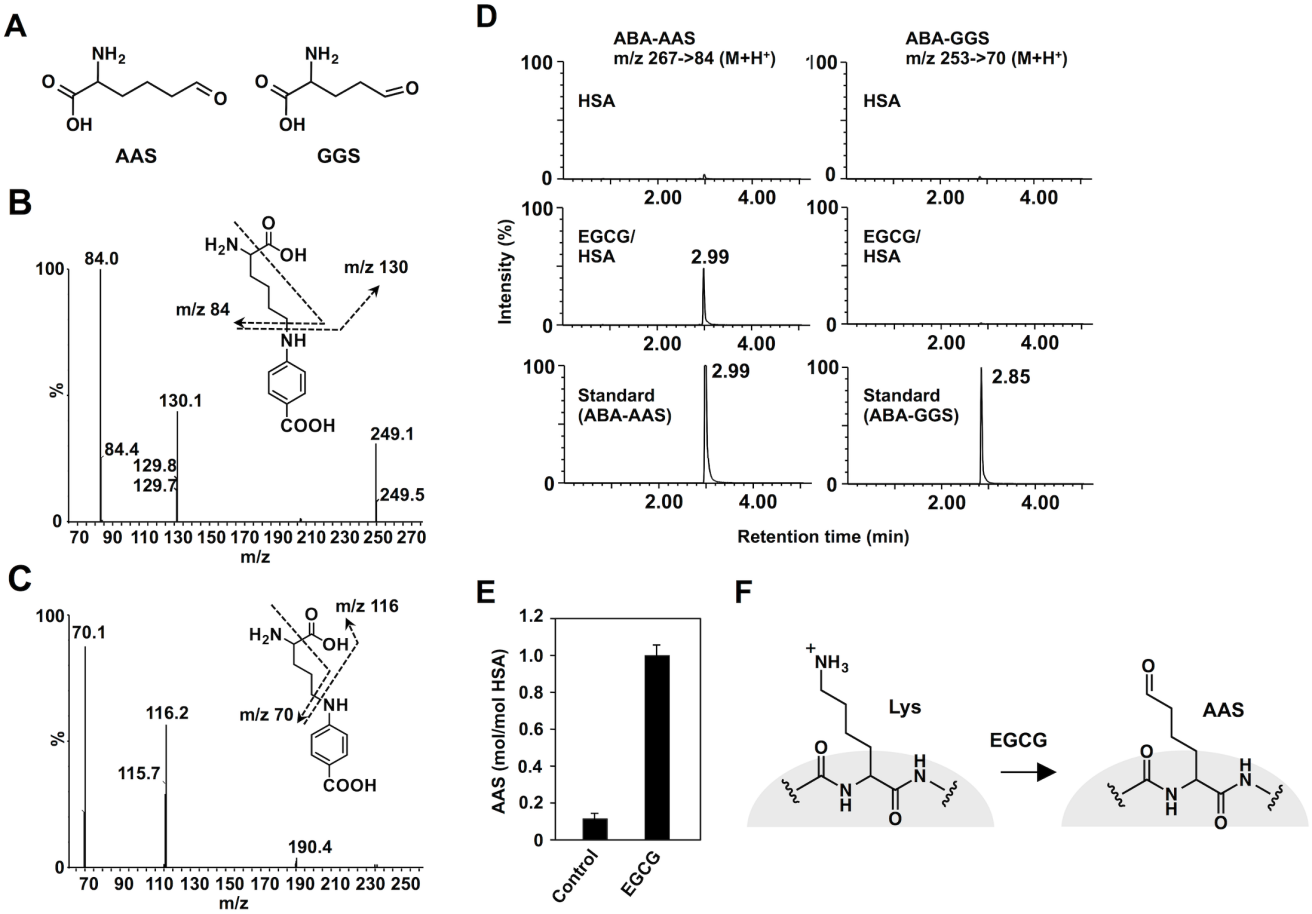
LC-ESI-MS/MS analysis of oxidized amino acids in the EGCG-treated HSA. (**A**) Chemical structures of aminoadipic semialdehyde (AAS) and glutamic semialdehyde (GGS). (**B**) Collision-induced dissociation of the [M+H]^+^ of ABA-AAS at *m/z* 267 at a collision energy of 25 V and the proposed structures of individual ions. (**C**) Collision-induced dissociation of the [M+H]^+^ of ABA-GGS at *m/z* 253 at a collision energy of 25 V and the proposed structures of individual ions. (**D**) The ion current tracings of ABA-AAS (*left three tracings*) and ABA-GGS (*right three tracings*) using LC-ESI-MS/MS with SRM. (**E**) Determination of AAS in the EGCG-treated HSA. HSA (1 mg/ml) was incubated with EGCG (1 mM) in 0.1 ml of PBS (pH 7.4) for 24 h at 37°C. The yield of AAS was semi-quantitatively determined based on a calibration curve (S3 Fig). (**F**) Schematic illustration of the transformation of a lysine residue to AAS by EGCG.

On the other hand, we characterized the oxidized amino acids generated via oxidative deamination by EGCG. To this end, we sought to detect aminoadipic semialdehyde (AAS) and γ-glutamic semialdehyde (GGS) (Fig 4A), the most predominant forms of the carbonyl amino acids [2], in the EGCG-treated HSA. The EGCG-treated and untreated HSAs were derivatized with *p*-aminobenzoic acid (ABA), hydrolyzed, and analyzed by LC-ESI-MS/MS in positive ion mode using MRM (Fig 4B and 4C). As shown in Fig 4D, one major product, which was inseparable from the ABA-AAS, appeared at 2.99 min in the EGCG-treated HSA. However, ABA-GGS was not detected in the control and EGCG-treated HSAs. Upon incubation of HSA with 1 mM EGCG for 24 h at 37°C, about 1 molecule of AAS per protein molecule was detected (Fig 4E, S3 Fig). Thus, it appeared that EGCG selectively acts on the lysine residues of the protein to generate AAS (Fig 4F). More interestingly, among phytochemicals tested, the oxidized lysine was detected only in the EGCG-treated HSA (S4 Fig).

### Identification of AAS Formation Sites and Molecular Modeling Studies

**Fig 5:**
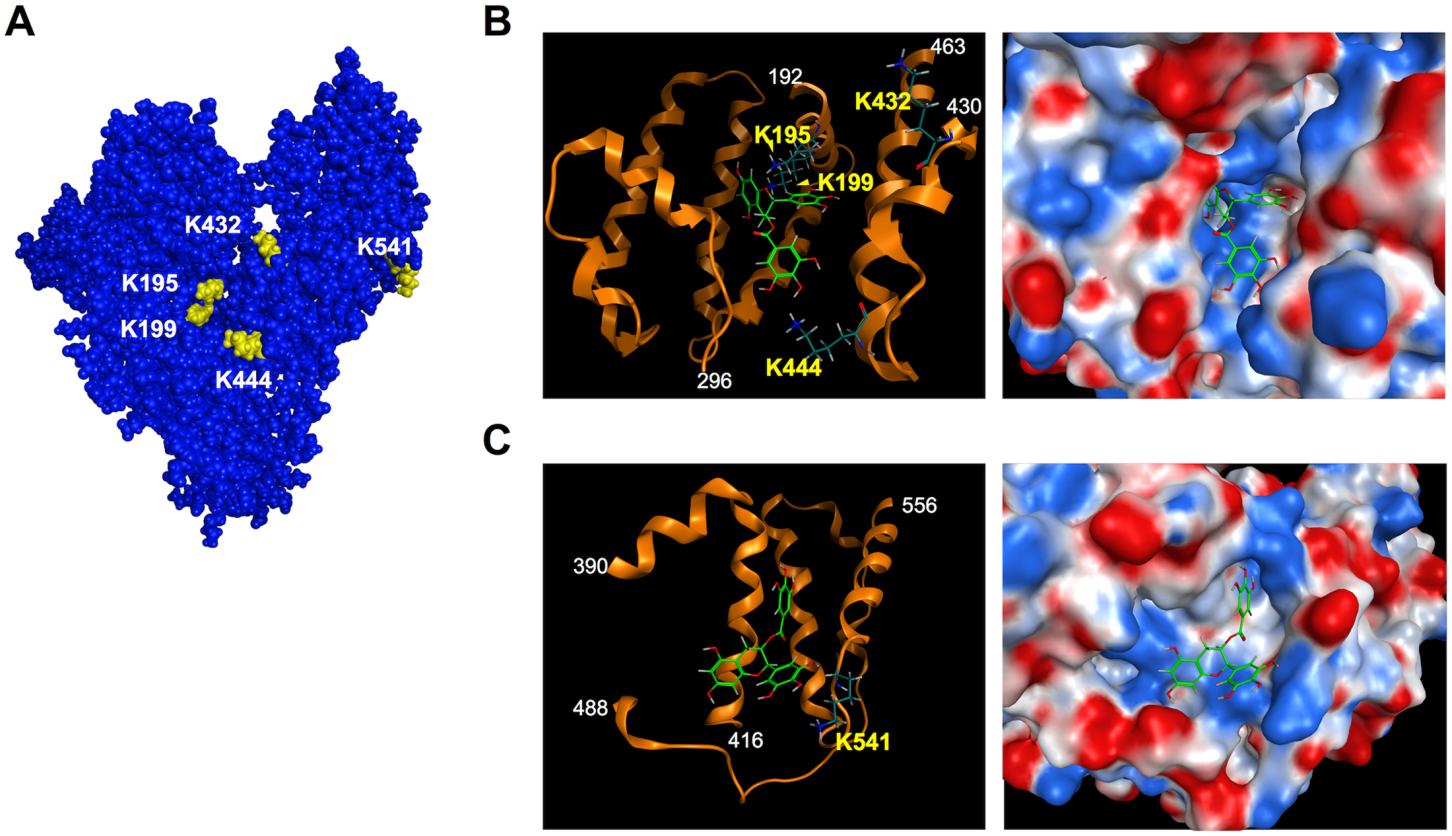
Docking simulations of EGCG to HSA. (**A**) Surface representation of HSA with the lysine residues sensitive to oxidation highlighted in yellow. (**B**) *Left*, schematic representation of the EGCG binding site in subdomains IIA and IIIA. Ribbon model is colored in orange. *Right*, close up view of the EGCG-binding pocket from its entrance located in subdomains IIA and IIIA. The electrostatic potential is represented on a color scale from blue for a positive potential, white for neutral, to red for a negative potential. (**C**) *Left*, schematic representation of the predicted EGCG binding site in subdomain IIIB. Ribbon model is colored in orange. *Right*, close up view of the predicted EGCG-binding pocket in subdomain IIIB. In panels **B** and **C**, selected residues shown in stick and color-coded by atom type: carbon in *dark green*; oxygen in *red*; and nitrogen in *blue*. The electrostatic potential is represented on a color scale from blue for a positive potential, white for neutral, to red for a negative potential. The EGCG molecule is shown in stick and color-coded by atom type: carbon in *right green*; oxygen in *red*.

To gain structural insight into the EGCG-oxidized HSA, we characterized the AAS formation sites in the EGCG-treated HSA using nano-LC-MALDI-TOF MS/MS and found that EGCG mediated the transformation of the lysine residues into AAS at Lys-195, 199, 432, 444 and 541 [S1 Table, S5–S9 Figs). The results indicated that the oxidative deamination mainly occurred at the lysine residues around the binding pocket of subdomains IIA and IIIA (Lys-195, 199, 432, 444) and subdomain IIIB (Lys-541) (Fig 5A–5C). Thus, it can be speculated that EGCG selectively oxidizes these lysine residues due to its high affinity to the binding sites.

**Fig 6:**
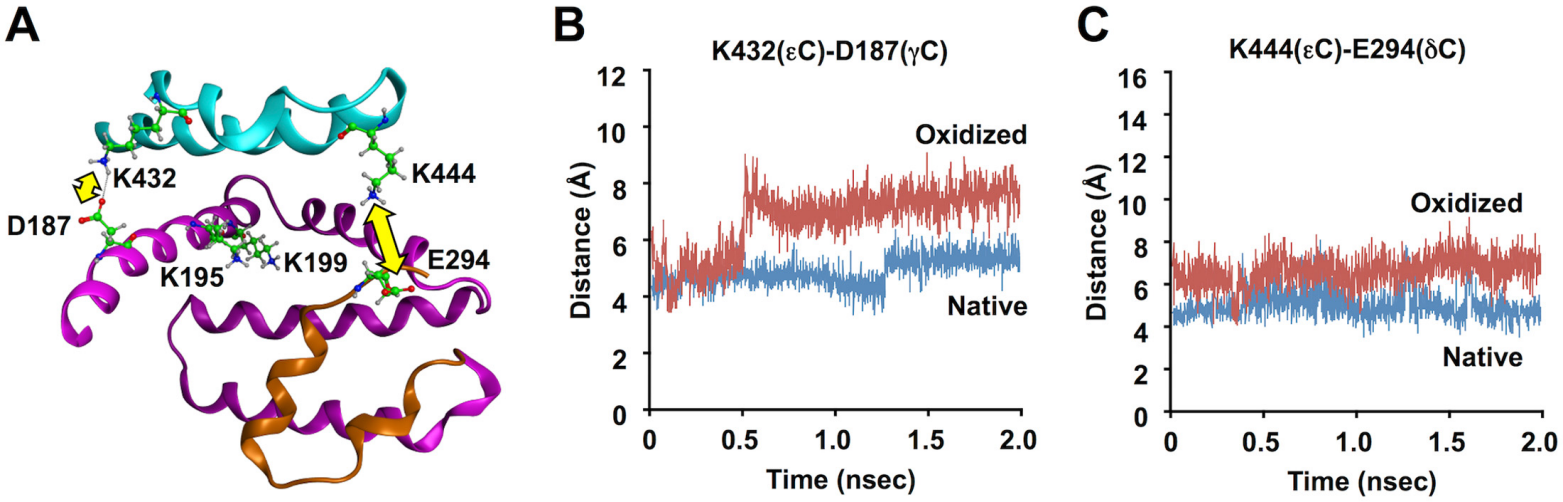
Changes in the electrostatic interaction of lysine residues as a consequence of oxidative deamination. (**A**) Schematic representation of the EGCG binding site in subdomains IIA and IIIA. Electrostatic interactions between Lys-432 and Asp-187 and between Lys-444 and Glu-292 are shown with yellow arrows. (**B**) Time dependent evolution of distance between the Lys-432 ε-carbon and the carboxyl group (γ-COOH) of Asp-187 before and after AAS formation at Lys-432. (**C**) Time dependent evolution of distance between the Lys-444 ε-carbon to the carboxyl group (δ-COOH) of Glu-292 before and after AAS formation at Lys-444.

A molecular dynamics simulation of HSA was further conducted to predict the structural impacts on the protein conformation as a consequence of the oxidative deamination of specific lysine residues. In the case of this study, we focused on the lysine residues, Lys-432 and 444, localized within subdomain IIIA due to the presence of potential electrostatic interactions involving these lysine residues. A molecular dynamics simulation indicated two pairs of electrostatic interactions between Lys-432 and Asp-187 and between Lys-444 and Glu-294 (Fig 6A); in the 2 ns simulation, the distance between the terminal carbon atoms in each amino-acid pair (Lys-432 (ε-CH_2_)-Asp-187 (γ-COOH) and Lys-444 (ε-CH_2_)-Glu-294 (δ-COOH)) was maintained within 6 Å (Fig 6B and 6C). The replacement of lysine residues at positions 432 and 444 by AAS resulted in the increasing distances between corresponding atoms, indicating that AAS formation and consequent loss of the electrostatic interactions lead to the dissociation of these pairs of amino acid residues. These data suggest that the transformation of the lysine residues to AAS might cause destabilization at the EGCG-binding site of the protein.

### Electronegative Potential of EGCG-Treated Proteins

**Fig 7:**
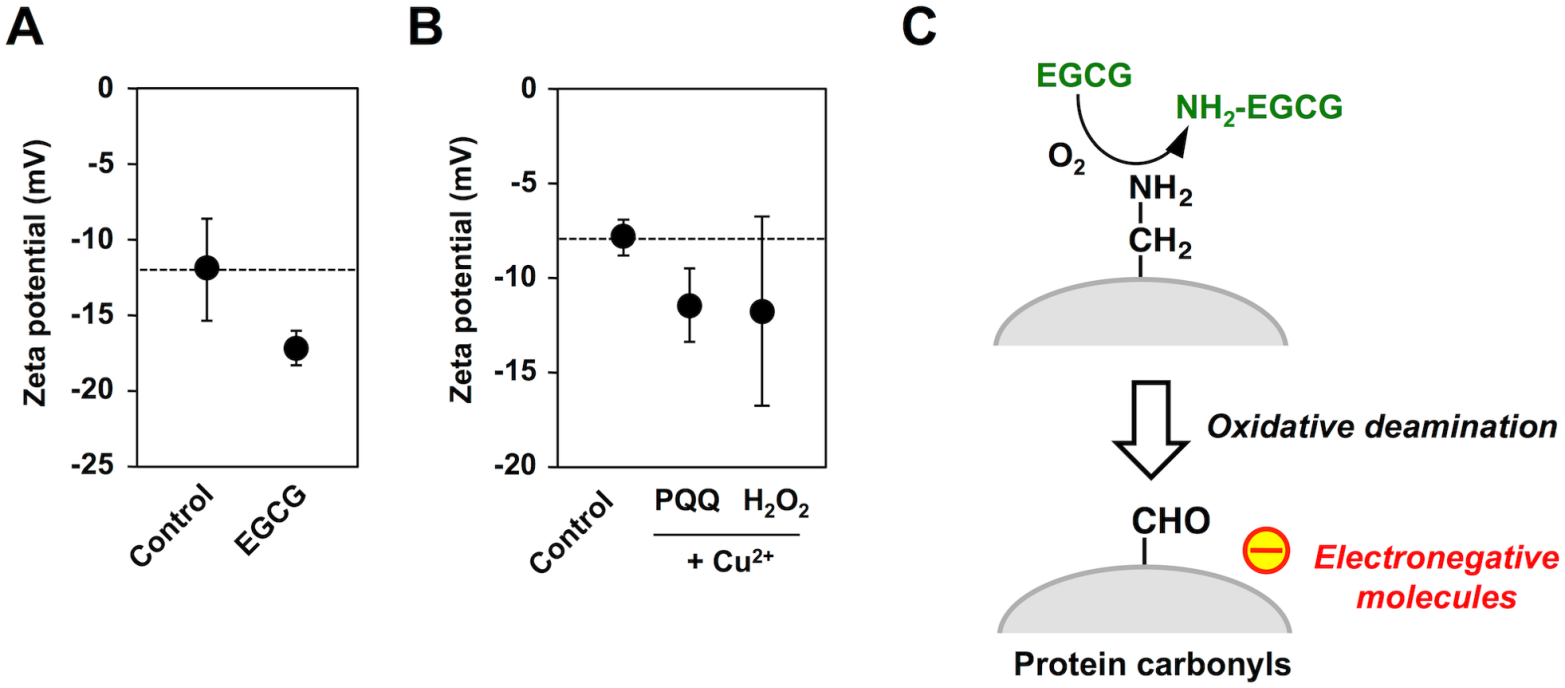
Formation of electrically-charged proteins by EGCG. (**A**) Changes in the zeta potential of HSA treated with the catechins. HSA (1 mg/ml) was incubated with 1 mM catechins in 0.1 ml of PBS (pH 7.4) for 24 h at 37°C. (**B**) Changes in the zeta potential of BSA treated with the metal-catalyzed oxidation reactions. BSA (1 mg/ml) was incubated with 200 μM PQQ or 1 mM H_2_O_2_ in the presence and absence of 100 μM Cu^2+^ in 0.1 ml of PBS (pH 7.4) for 24 h at 37°C. (**C**) Schematic illustration of the EGCG-mediated transformation of HSA into electronegative molecules via oxidative deamination.

Because the oxidative deamination is an amino charge-disappearing reaction, we speculated that the protein carbonyl formation by EGCG might represent the generation of electronegative proteins. Hence, an electrochemical property of the EGCG-treated protein was evaluated by measuring the zeta potential. As shown in Fig 7A, the zeta potential of HSA decreased to approximately -17 mV upon EGCG treatment. A similar decrease in the zeta potential was also observed in the metal-catalyzed oxidation of the protein by pyrroloquinoline quinone (PQQ) or H_2_O_2_ (Fig 7B). These data implicate that EGCG treatment results in the increased negative charge of the protein due to the oxidative deamination of the lysine residues (Fig 7C).

### Recognition of EGCG-Oxidized Proteins by IgM

**Fig 8:**
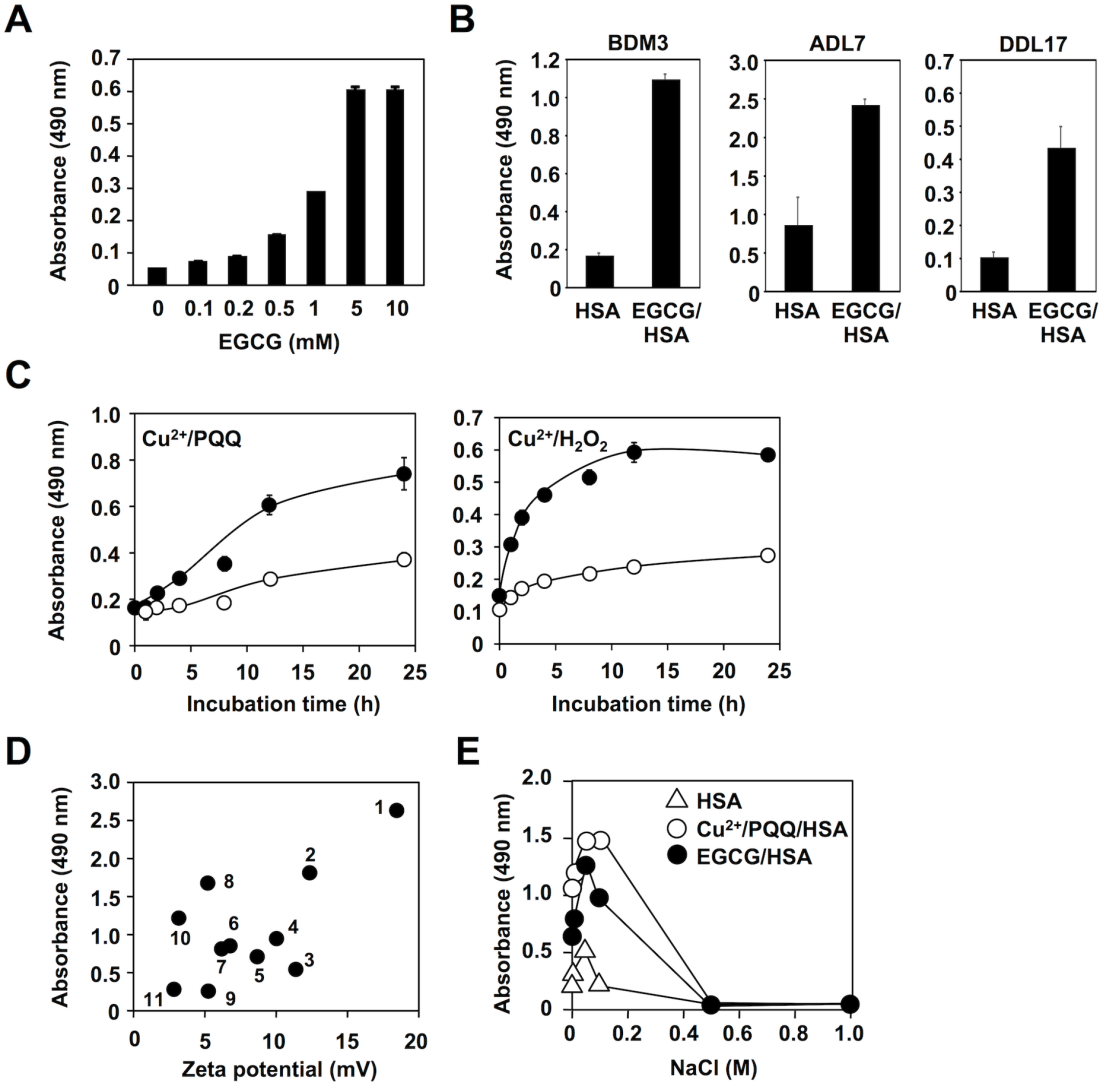
EGCG as a natural source of innate epitopes. (**A**) Dose-dependent transformation of HSA into innate epitopes by EGCG. HSA (1 mg/ml) was incubated with EGCG (0–10 mM) in 0.1 ml of PBS (pH 7.4) for 24 h at 37°C. Cross-reactivity with the IgM mAb BDM1 was examined by ELISA. (**B**) HSA (1 mg/ml) was incubated with EGCG (1 mM) in 0.1 ml of PBS (pH 7.4) for 24 h at 37°C. Cross-reactivity with the IgM mAbs, BDM3, ADL7, and DDL 17, was examined by ELISA. (**C**) Transformation of serum albumins into the innate epitopes during the Cu^2+^-catalyzed oxidation reactions. *Left panel*: Cu^2+^/PQQ. *Right panel*: Cu^2+^/H_2_O_2_. BSA (1 mg/ml) was incubated with 200 μM PQQ or 1 mM H_2_O_2_ in the presence and absence of 100 μM Cu^2+^ in 0.1 ml of PBS (pH 7.4) at 37°C. Cross-reactivity with the sera from the MRL-MpJ (*open circle*) and MRL-lpr (*closed circle*) mice was examined by ELISA. (**D**) Correlation between electronegativity and innate epitope potential of the polyphenol-treated proteins. BSA (1 mg/ml) was incubated with 1 mM polyphenols in 0.1 ml of PBS (pH 7.4) for 24 h at 37°C. An electrochemical property of the polyphenol-treated proteins was evaluated by measuring the zeta potential. An innate epitope potential (IgM titer) of the polyphenol-treated proteins was evaluated by their cross-reactivity with the sera from the MRL-*lpr* mice. *Numbers*: *1*, cyanidin; *2*, malvidin; *3*, diosmetin; *4*, delphinidin; *5*, eriodictiol; *6*, ellagic acid; 7, caffeic acid; *8*, piceatannol; *9*, quercetin; *10*, gallic acid; *11*, control. (**E**) Effect of NaCl on binding of the mAb BDM1 to the antigens. The mAb was preincubated by serial dilutions of NaCl before addition to the ELISA plates coated with the native HSA (*open triangle*), Cu^2+^/PQQ-treated HSA (*open circle*), and EGCG-treated HSA (*closed circle*).

We have previously shown that the electronegative potential of modified self-proteins, such as advanced glycation end products (AGEs) and oxidized LDL, is involved in the multi-specificities of the IgM mAbs [9]. Hence, we tested whether the natural Abs could recognize the oxidized serum albumins. As expected, EGCG dose-dependently generated the innate epitopes recognized by the IgM mAb BDM1 (Fig 8A). We also tested several other IgM mAbs that cross-react with multiple antigens, such as AGEs and oxidized LDL, and observed that they commonly recognized the EGCG-treated HSA as an epitope (Fig 8B). In addition, our preliminary study showed that normal human serum cross-reacted with the EGCG-treated HSA slightly greater than control HSA (S10 Fig). These data and the observations that the metal-catalyzed oxidation of the albumin by H_2_O_2_ or PQQ transformed the protein into the innate epitopes (Fig 8C) suggest that protein carbonylation may represent a key mechanism for the formation of innate epitopes. We also observed that several polyphenols gave rise to a significant decrease in the zeta potential of the protein, which was well correlated with the cross-reactivity involving the sera from the MRL-*lpr* mice, a spontaneous murine model of autoimmune disease (Fig 8D). In addition, we evaluated the involvement of the electric interaction between the Ab and the EGCG-treated HSA by manipulation of the ionic strength and observed that NaCl inhibited the binding of the IgM mAb, showing specificity toward multiple electronegative molecules, to the antigens in a dose-dependent manner (Fig 8E).

### A Functional Determinant for the Formation of Innate Epitopes by EGCG

**Fig 9:**
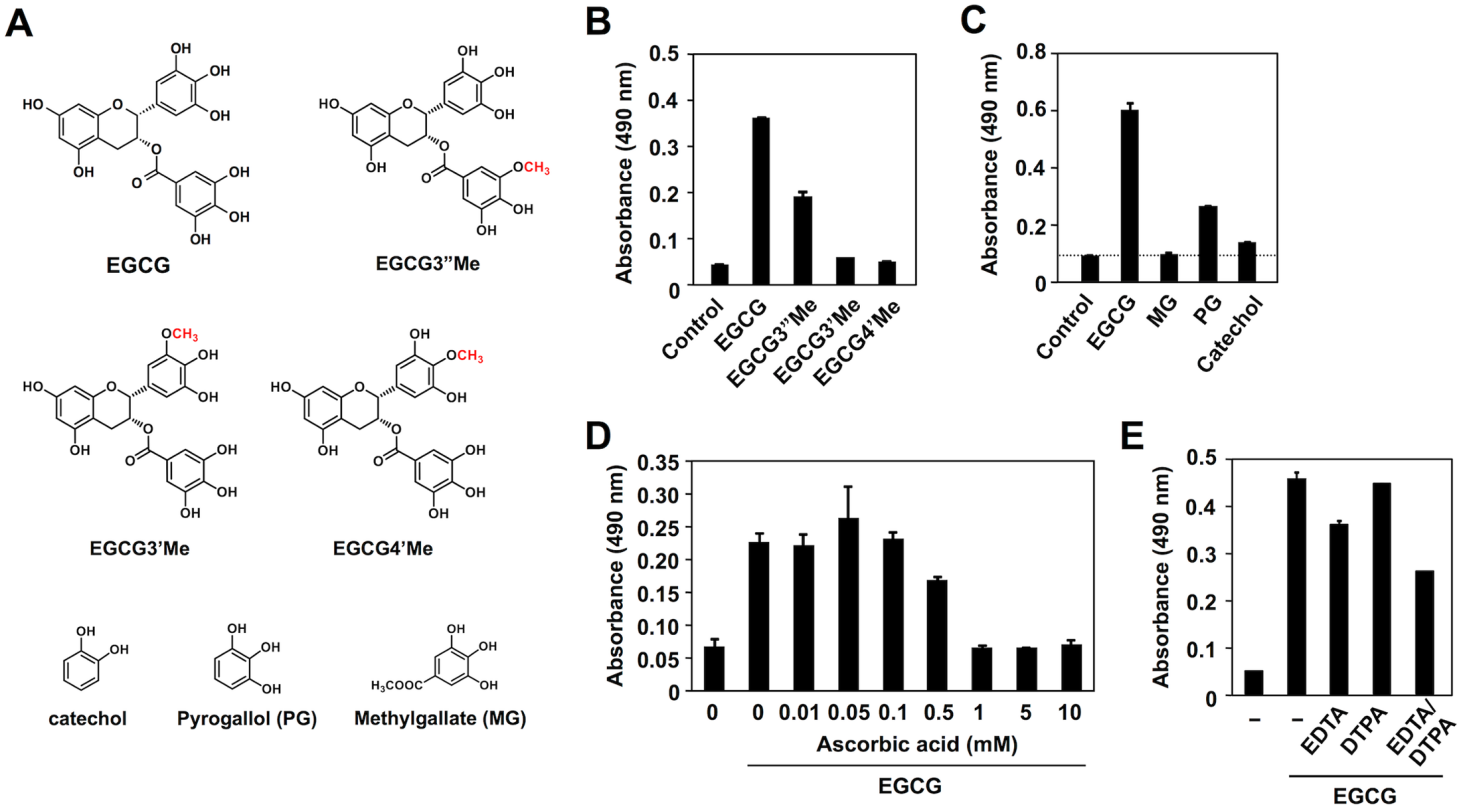
A functional determinant for the formation of innate epitopes by EGCG. (**A**) Chemical structures of EGCG, *O*-methyl derivatives of EGCG, and the structural elements of EGCG. **(B)** Transformation of HSA into the innate epitopes by *O*-methyl derivatives of EGCG. (**C**) Transformation of HSA into the innate epitopes by the structural elements of EGCG. In panels **B** and **C**, HSA (1 mg/ml) was incubated with 1 mM EGCG and its related compounds in 0.1 ml of PBS (pH 7.4) for 24 h at 37°C. (**D**) Dose-dependent inhibition of the EGCG-mediated transformation of HSA into the innate epitopes by ascorbic acid. HSA (1 mg/ml) was incubated with EGCG (1 mM) in the presence and absence of ascorbic acid (0–10 mM) in 0.1 ml of PBS (pH 7.4) for 24 h at 37°C. (**E**) Effect of metal chelators on the EGCG-mediated transformation of HSA into the innate epitopes. HSA (1 mg/ml) was incubated with 1 mM EGCG in the presence and absence of 100 μM chelators in 0.1 ml of PBS (pH 7.4) for 24 h at 37°C. In panels **B**-**E**, cross-reactivity of the protein with the IgM mAb BDM1 was examined by ELISA.

To gain insight into structure-activity relationships of EGCG for the formation of innate epitopes, we synthesized several *O*-methyl derivatives of EGCG (Fig 9A) and measured their abilities to form innate ligands upon reaction with HSA. Although the *O*-methylation of the 3''-position in the galloyl moiety (EGCG3”Me) still retains 50% of the original activity, the *O*-methylation of the 3’- or 4’-position in the pyrogallol moiety of EGCG (EGCG3’Me and EGCG4’Me) abolishes or weakens the activity (Fig 9B). In addition, among the structural elements of EGCG, namely catechol, pyrogallol and methylgallate, only pyrogallol generated the epitopes (Fig 9C), suggesting that the pyrogallol moiety, which is highly sensitive to oxidation, might represent the functional determinant for formation of the innate epitopes. These results and the observation that the EGCG-mediated formation of innate epitopes was inhibited by ascorbic acid (vitamin C) (Fig 9D) suggest that the oxidation of EGCG might be essential for the formation of epitopes. However, the metal-chelating agents, EDTA and DTPA, showed little or no effect (Fig 9E). Thus, it is likely that EGCG may undergo auto-oxidation and generate innate epitopes on the protein by a metal-independent mechanism.

## Discussion

Protein carbonyls have become a commonly used biomarker of protein oxidation in cells and tissues and have been detected in the tissues from age-related pathologies, such as atherosclerosis, neurodegenerative disorders, and cataracts [1]. Carbonyl groups can be introduced into proteins through a variety of mechanisms [1]. Stadtman initially proposed that a metal-catalyzed oxidation might be the most important mechanism for the formation of protein carbonyls [21]. This process includes the generation of H_2_O_2_ and reduction of Fe^3+^ or Cu^2+^ by a suitable electron donor; Fe^2+^ and Cu^+^ ions bind to specific metal binding sites on proteins and react with H_2_O_2_ to generate reactive oxygen species, leading to the oxidation of amino acid residues to generate the oxidized products, such as AAS and GGS, with a carbonyl functionality [2, 22]. In addition to the metal-catalyzed oxidation of proteins, the protein carbonyls can be generated through the covalent binding of reactive aldehyde species to proteins [23–25]. It has been suggested that the carbonylated proteins play a major role in a number of human diseases and aging. However, little was known about the biological significance of the formation of protein carbonyls. We have now established that the oxidized proteins with the carbonyl functionality and electronegative potentials could be a common target of IgM Abs. This finding and the apparent presence of protein carbonyls *in vivo* provide a possible link connecting oxidative protein modification and innate immunity. This finding also led us to speculate that the protein carbonyls might constitute a previously unrecognized, but important class of innate ligands recognized by the pattern recognition receptors.

In the present study, we established that EGCG selectively mediated the oxidative deamination of lysine residues in proteins. The oxidative deamination activity of EGCG was initially proposed by Akagawa et al. [10, 26], in which oxidized EGCG, generating *O*-quinones, might covalently bind primary amines to form Schiff-base intermediates, such as iminoquinone and iminophenol, followed by the conversion of the intermediates to oxidatively deaminated products (aldehydes). Our click chemistry-based study unequivocally revealed the binding of the EGCG probe to the protein and demonstrated that the protein-bound EGCG was generated independent of the cysteine residue (Fig 1B–1D). These findings suggested that a Schiff-base intermediate between the oxidized EGCG and lysine residues might be relatively stable. The formation of a putative Schiff base intermediate was correlated with the production of the oxidized lysine (AAS) in the EGCG-treated HSA. In addition, using LC-ESI-MS, we successfully detected both the oxidized and aminated EGCG as the products of the EGCG-mediated oxidative deamination (Fig 2). More strikingly, the aminated EGCG was also detected in the sera of the BALB/C mice treated with EGCG (Fig 3). These data provide the first mechanistic details of the oxidative deamination activity of EGCG.

To gain structural insight into the EGCG-mediated oxidative modification of HSA, we sought to detect the oxidized amino acids, AAS and GGS, using LC-ESI-MS/MS. However, it appeared that the formation of protein carbonyls by EGCG was ascribed to the selective oxidation of the lysine residues to generate AAS. The yield of AAS, when the HSA was incubated with 1 mM EGCG for 24 h at 37°C, was about 1 molecule of AAS per protein molecule (Fig 4). Moreover, using nano-LC-MALDI-TOF MS/MS, we identified five lysine residues (Lys-195, 199, 432, 444 and 541) as the AAS formation sites (Fig 5, S1 Table, S5–S9 Figs). These lysine residues are located in subdomain IIA (Lys-195 and Lys-199), subdomain IIIA (Lys-432 and Lys-444), and subdomain IIIB (Lys-541). Subdomains IIA and IIIA, in particular, have a pocket formed of hydrophobic and positively charged amino acid residues and can bind a wide range of compounds. Docking studies indeed showed that the hydrophobic pocket at subdomains IIA and IIIA is large enough to be involved in the binding of EGCG (Fig 5). The result agrees with the previous finding that EGCG binds to residues located in subdomains IIA and IIIA [27]. The *in silico* experiment revealed several docking poses of EGCG in the pocket, some of which can account for the proximity between the four lysine residues around the pocket and the galloyl groups of EGCG and its resulting oxidation. In addition, based on the identification of Lys-541 as the AAS formation site, molecular modeling also revealed the presence of a potential EGCG-binding pocket in subdomain IIIB. We also conducted molecular dynamics simulation of the oxidatively deaminated HSA to predict structural changes in the protein conformation and observed that the AAS formation on the lysine residues, Lys-432 and Lys-444, in subdomain IIIA revealed a remarkable change in the electrostatic interaction with the spatially proximate acidic amino acid residues (Fig 6). Previous studies have shown that oxidation of HSA modifies the conformational stability of the protein structure, leading to protein compaction, increased thermal stability, and reduced association propensity [28,29]. Thus, the transformation of the lysine residues involved in the interaction with EGCG into the oxidized lysine causes loss of the electrostatic interactions essential for maintaining the higher order structure of HSA at the ligand binding-site that may result in global changes in the conformation of the protein and in the disruption of the interaction critical for the high affinity binding of ligands. This may be associated with the consequences of the production of innate epitopes.

Natural IgM Abs are present in the circulation of normal humans and other mammalian species. They are mainly produced by B1 lymphocytes in the absence of external antigen stimulation at tightly regulated levels, providing immediate protection against pathogens and play an important role in the host defense mechanism against various stresses. They are commonly multi-specific and recognize specific molecular patterns with no apparent structural homology [30–32]. Previous studies have shown that the modified self-proteins, such as oxidized low-density lipoproteins (oxidized LDL), are important targets of the natural Abs [33, 34]. These modified proteins have several characteristics that are distinct from native proteins, including an increased negative charge due to modification of the basic amino acid residues. In our previous study, we proposed a theory for the involvement of electronegative potentials in the multi-specificity of the natural Abs [9]. This hypothesis was based on the finding that the IgM mAbs recognized a variety of electronegative molecules, including the advanced glycation end products (AGEs), oxidized LDL, and nucleic acids. Consistent with this hypothesis, the current study demonstrated the decrease in the zeta potential of the protein upon treatment with EGCG (Fig 7). In addition, IgM mAbs showed significant cross-reactivity to the EGCG-treated HSA (Fig 8). To the best of our knowledge, this is the first study reporting that phytochemicals transform self-proteins into the innate antigens. It is likely that EGCG gives rise to the increased negative charge of the protein probably due to the oxidative deamination of lysine residues and that the Abs may recognize these electrically-transformed proteins. These results are consistent with the fact that EGCG offers many health benefits from cancer prevention to helping prevent diabetes and heart disease. Our studies highlight the key role of EGCG in mediating the production of innate epitopes that could be an important trigger of the innate immune response. These findings suggest that the beneficial effects of polyphenols might be, at least in part, the result of triggering the immune response via the interaction with the serum albumins.

In our preliminary experiments, we observed that standardized green tea polyphenols, when administered in drinking water, mediated a modest increase in the serum IgM levels. In addition, although it was not statistically significant, the green tea-administered mice showed slightly higher IgG and IgM titers to the oxidized serum albumins compared to the control mouse (Hatasa, Y., Shibata, T., Tachibana, H., Uchida, K., unpublished observation). These data and the previous findings that the accumulation of lipid peroxidation-modified proteins in animal brains during aging could be reduced by the administration of EGCG [35, 36] emphasize the relevance of a tea-drinking habit for health. Although the potential role of tea polyphenols in health and disease prevention needs to be further explored, the interaction between catechins and serum albumins may not simply be the binding of exogenous small molecules by the proteins, but a possible trigger of immune response via an oxidative deamination of lysine residues. These findings offer an attractive hypothesis that phytochemicals and their metabolites possessing a similar oxidative deamination activity could be an important trigger of innate immune response, thereby contributing to the protection against exogenous pathogens and damage-associated molecules.

## Supporting Information

S1 Fig Collision-induced dissociation of the [M+H]+ of EGCG at m/z 459 (A) and the [M+H]+ of a product generated in the reaction of HSA with EGCG at m/z 458 (B) at a collision energy of 20V and the proposed structures of individual ions. (PDF)

S2 Fig LC-ESI-MS/MS analysis of EGCG-derived products generated upon incubation with HSA or ammonia. EGCG (1 mM) was incubated with HSA (1 mg/ml) or ammonia (0.1 mM) in PBS (pH 7.4) for 1 h. After removing proteins by precipitation with cold acetone, the samples were analyzed by LC-ESI-MS/MS with SRM mode.
(PDF)

S3 Fig Calibration curve for determination of AAS. Stoichiometry between the concentrations of ABA-AAS and the increase in peak area of the products showed a linear correlation.
(PDF)

S4 Fig Transformation of lysine residues into AAS by phytochemicals. HSA (1 mg/ml) was incubated with the phytochemicals (1 mM) in 0.1 ml of PBS (pH 7.4) for 24 h at 37°C. The treated and untreated HSAs were derivatized with ABA, hydrolyzed, and analyzed by LC-ESI-MS/MS in positive ion mode using MRM.
(PDF)

S5 Fig MS/MS spectrum of the ABA-AAS-containing HSA peptide. (PDF)

S6 Fig MS/MS spectrum of the ABA-AAS-containing HSA peptide. (PDF)

S7 Fig MS/MS spectrum of the ABA-AAS-containing HSA peptide. (PDF)

S8 Fig MS/MS spectrum of the ABA-AAS-containing HSA peptide. (PDF)

S9 Fig MS/MS spectrum of the ABA-AAS-containing HSA peptide. (PDF)

S10 Fig Cross-reactivity of HSA (*open circle*) and EGCG-treated HSA (*closed circle*) with the IgM of human serum was examined by ELISA. Normal human serum was used as the first antibody, and HRP-conjugated anti-human IgM antibody was used as the second antibody.
(PDF)

S1 Table Peptides identified by nano-LC-ESI-MS/MS from the EGCG-treated HSA. (PDF)
