# Supplementary material for: Oxidative Deamination of Serum Albumins by (-)-Epigallocatechin-3-O-Gallate: A Potential Mechanism for the Formation of Innate Antigens by Antioxidants
Source: PLoS One. 2016 Apr 5;11(4):e0153002. doi: 10.1371/journal.pone.0153002 (PMC4821561; doi:10.1371/journal.pone.0153002)
Supplement: S1 Fig — (PDF) [file pone.0153002.s001.pdf]

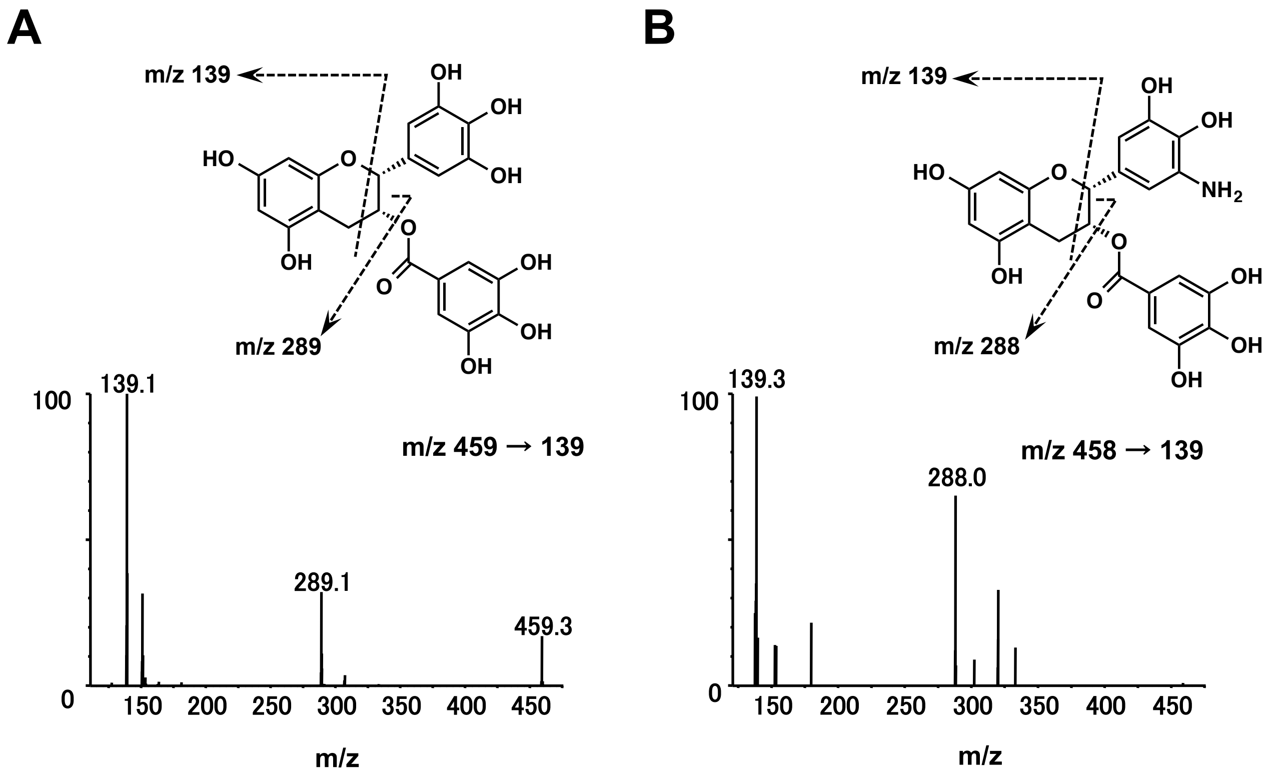

Fig. S1. Collision-induced dissociation of the $[M+H]^+$ of EGCG at *m/z* 459 (A) and the $[M+H]^+$ of a product generated in the reaction of HSA with EGCG at *m/z* 458 (B) at a collision energy of 20 V and the proposed structures of individual ions.
