# Supplementary material for: Oxidative Deamination of Serum Albumins by (-)-Epigallocatechin-3-O-Gallate: A Potential Mechanism for the Formation of Innate Antigens by Antioxidants
Source: PLoS One. 2016 Apr 5;11(4):e0153002. doi: 10.1371/journal.pone.0153002 (PMC4821561; doi:10.1371/journal.pone.0153002)
Supplement: S2 Fig — EGCG (1 mM) was incubated with HSA (1 mg/ml) or ammonia (0.1 mM) in PBS (pH 7.4) for 1 h. After removing proteins by precipitation with cold acetone, the samples were analyzed by LC-ESI-MS/MS with SRM mode. (PDF) [file pone.0153002.s002.pdf]

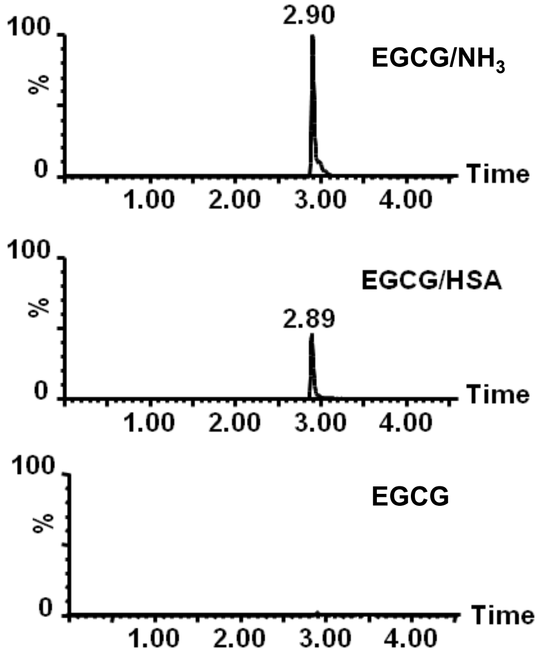

Fig. S2. LC-ESI-MS/MS analysis of EGCG-derived products generated upon incubation with HSA or ammonia.

EGCG (1 mM) was incubated with HSA (1 mg/ml) or ammonia (0.1 mM) in PBS (pH 7.4) for 1 h. After removing proteins by precipitation with cold acetone, the samples were analyzed by LC-ESI-MS/MS with SRM mode.
