# Supplementary material for: Oxidative Deamination of Serum Albumins by (-)-Epigallocatechin-3-O-Gallate: A Potential Mechanism for the Formation of Innate Antigens by Antioxidants
Source: PLoS One. 2016 Apr 5;11(4):e0153002. doi: 10.1371/journal.pone.0153002 (PMC4821561; doi:10.1371/journal.pone.0153002)
Supplement: S4 Fig — HSA (1 mg/ml) was incubated with the phytochemicals (1 mM) in 0.1 ml of PBS (pH 7.4) for 24 h at 37°C. The treated and untreated HSAs were derivatized with ABA, hydrolyzed, and analyzed by LC-ESI-MS/MS in positive ion mode using MRM. (PDF) [file pone.0153002.s004.pdf]

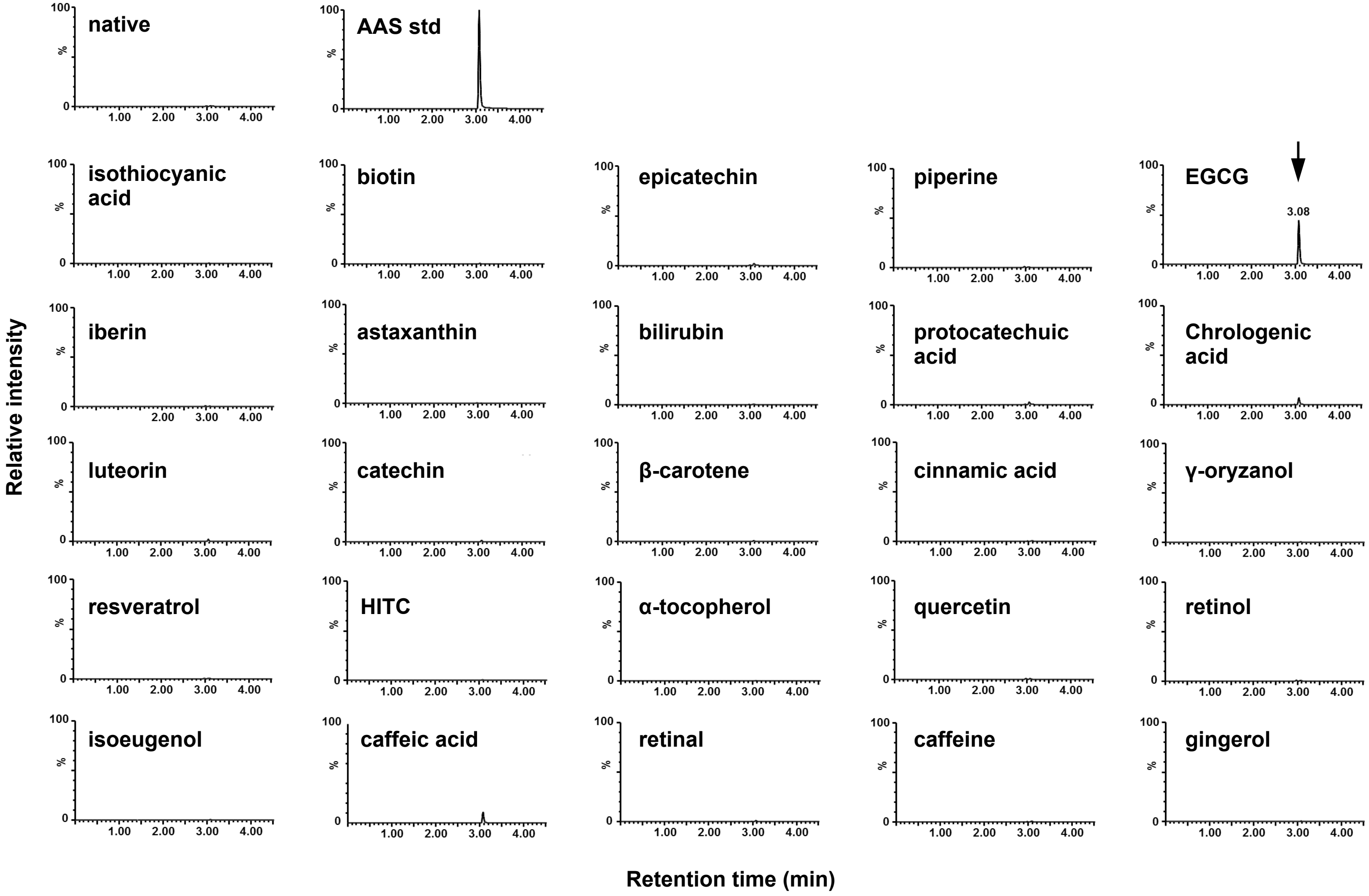

Fig. S4. Transformation of lysine residues into AAS by phytochemicals.

HSA (1 mg/ml) was incubated with the phytochemicals (1 mM) in 0.1 ml of PBS (pH 7.4) for 24 h at 37 °C. The treated and untreated HSAs were derivatized with ABA, hydrolyzed, and analyzed by LC-ESI-MS/MS in positive ion mode using MRM.
