# Supplementary figures and images for: Oxidative Deamination of Serum Albumins by (-)-Epigallocatechin-3-O-Gallate: A Potential Mechanism for the Formation of Innate Antigens by Antioxidants
Source: PLoS One. 2016 Apr 5;11(4):e0153002. doi: 10.1371/journal.pone.0153002 (PMC4821561; doi:10.1371/journal.pone.0153002)

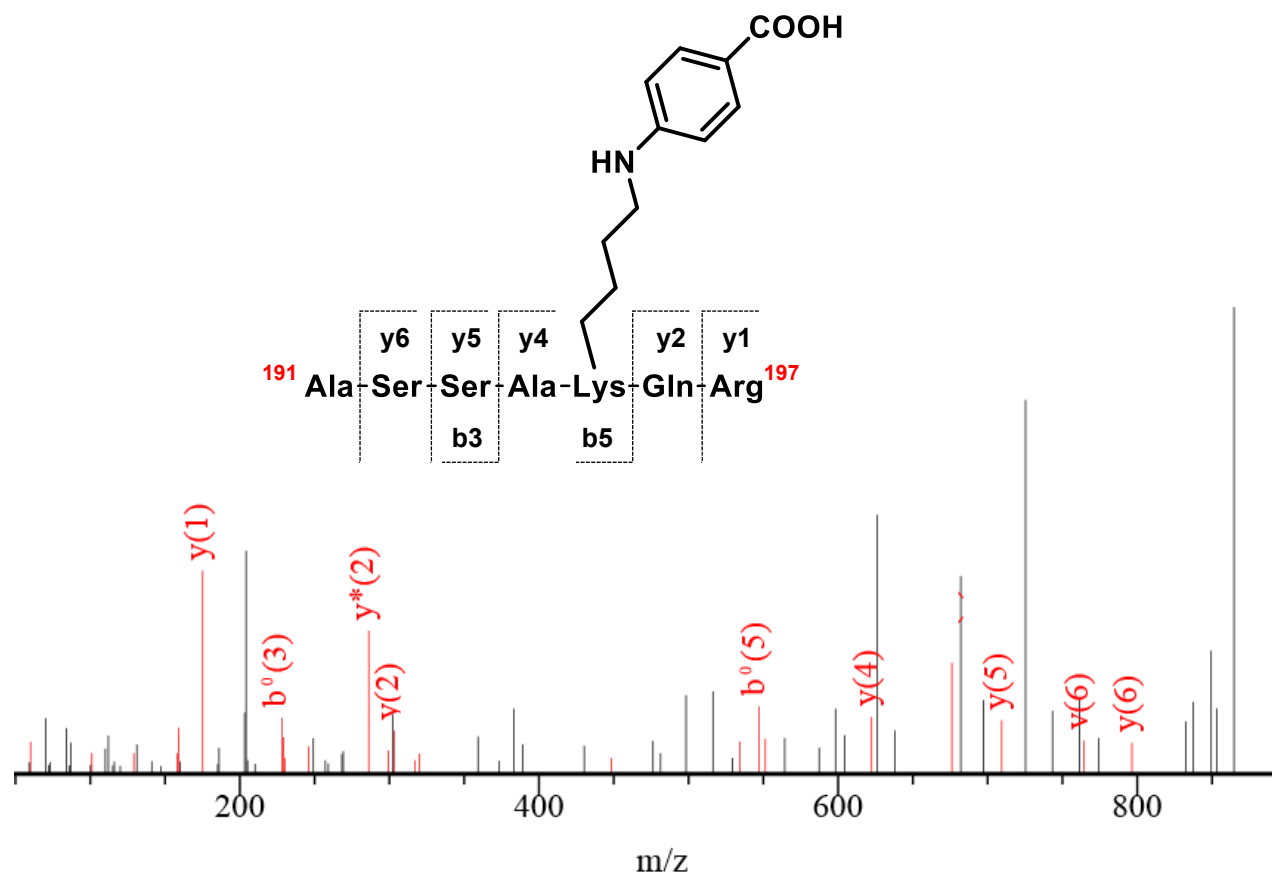

**Fig. S5. MS/MS spectrum of the ABA-AAS-containing HSA peptide.**

Supplement: S5 Fig — (PDF) [file pone.0153002.s005.pdf]

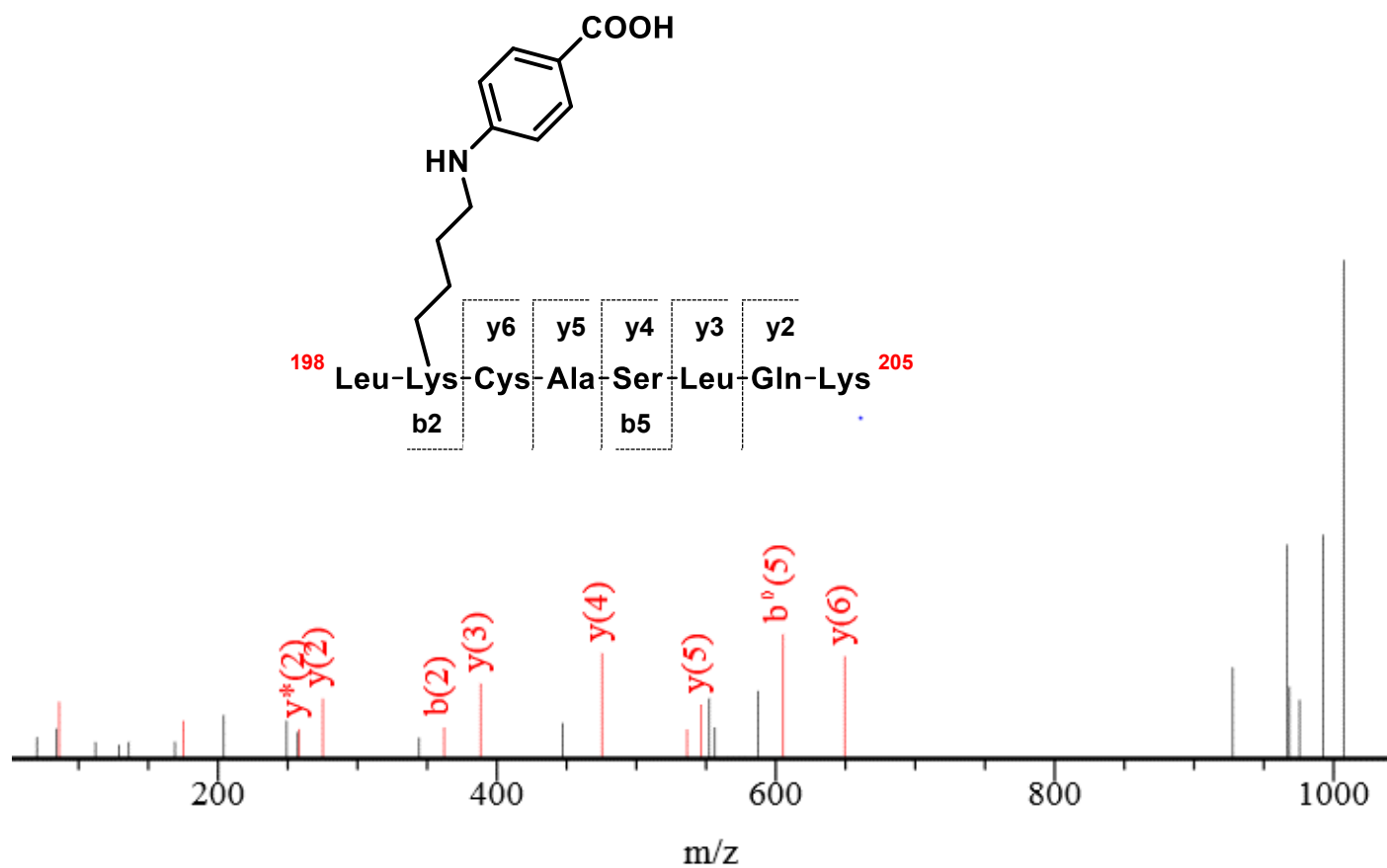

**Fig. S6. MS/MS spectrum of the ABA-AAS-containing HSA peptide.**

Supplement: S6 Fig — (PDF) [file pone.0153002.s006.pdf]

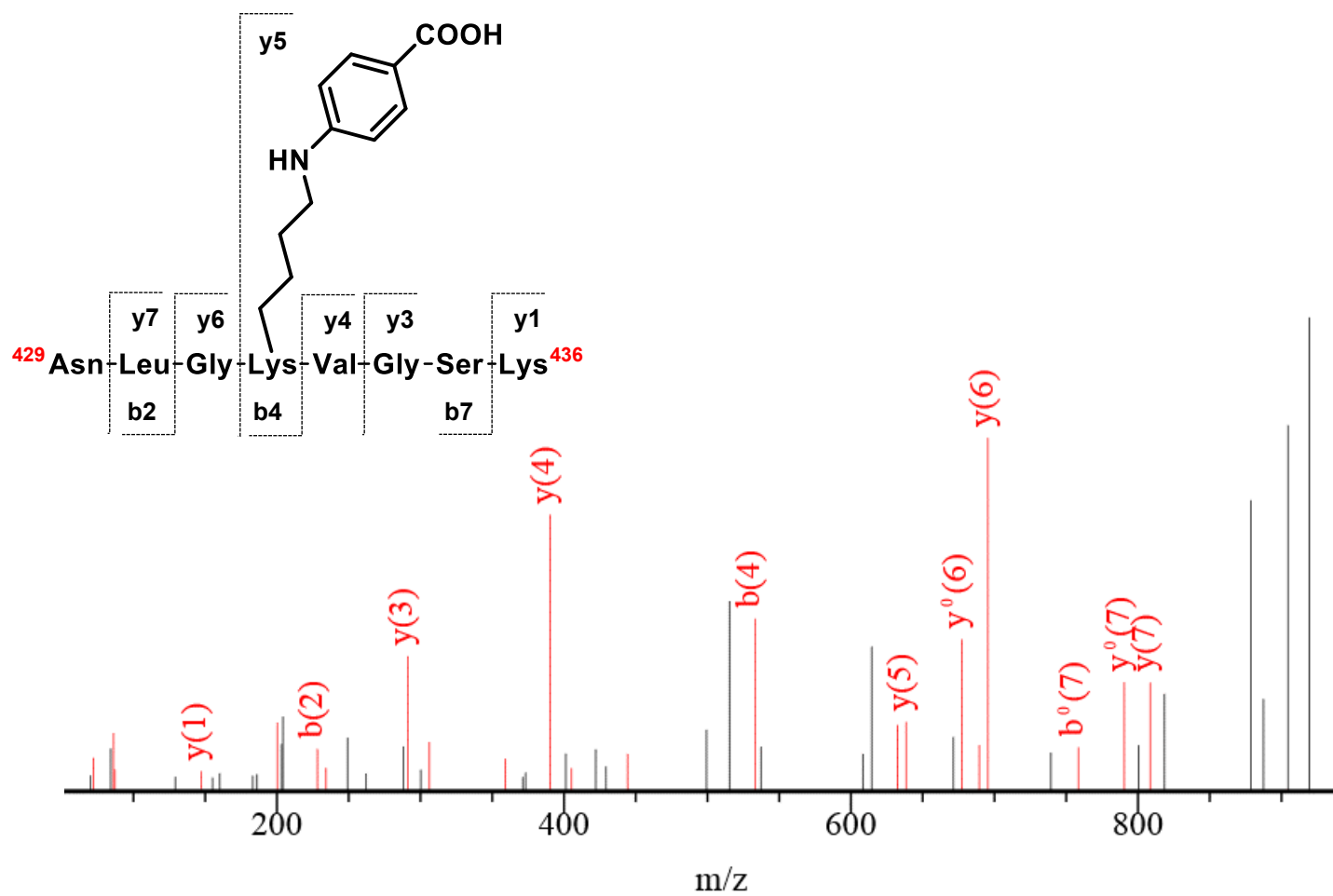

**Fig. S7. MS/MS spectrum of the ABA-AAS-containing HSA peptide.**

Supplement: S7 Fig — (PDF) [file pone.0153002.s007.pdf]

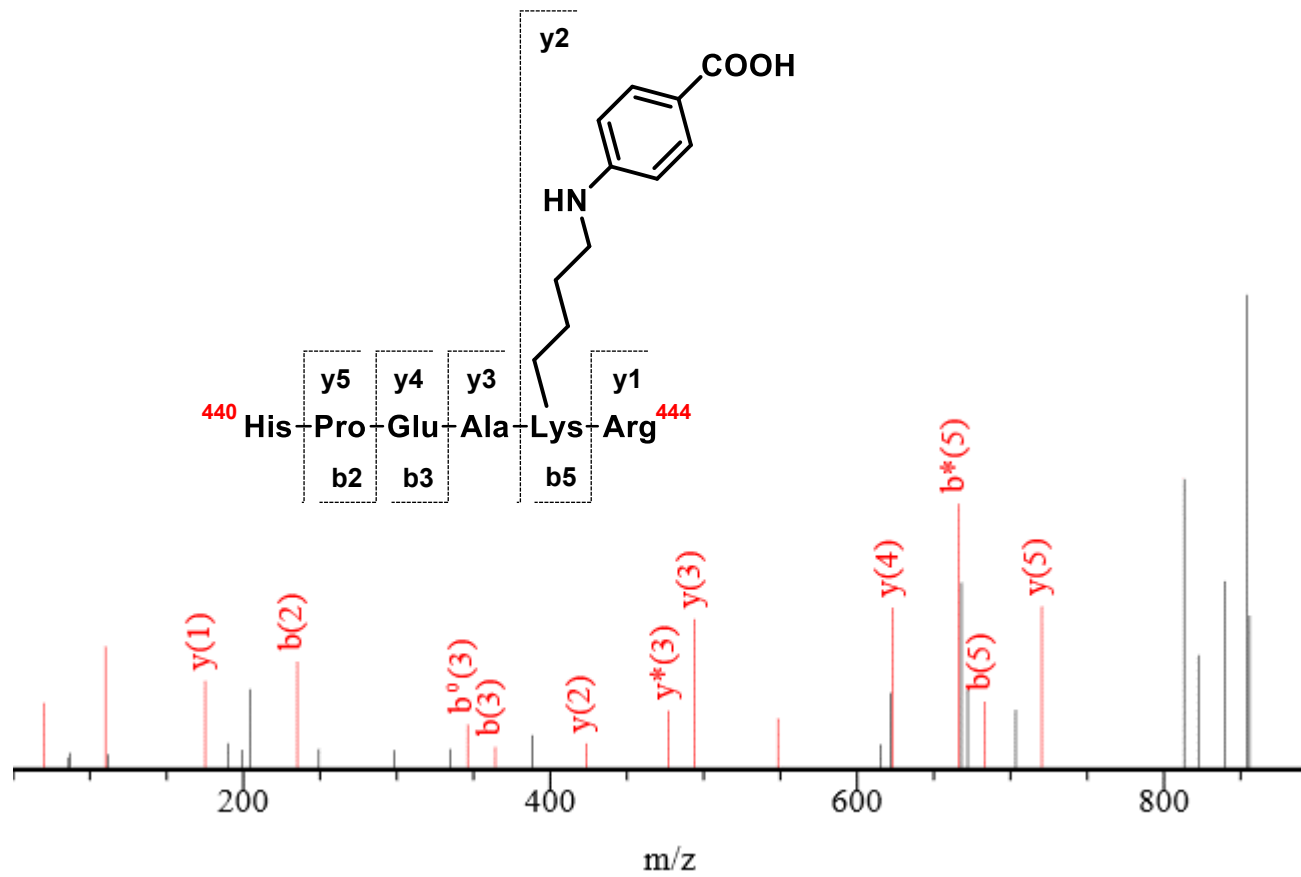

**Fig. S8. MS/MS spectrum of the ABA-AAS-containing HSA peptide.**

Supplement: S8 Fig — (PDF) [file pone.0153002.s008.pdf]
