# Supplementary material for: Oxidative Deamination of Serum Albumins by (-)-Epigallocatechin-3-O-Gallate: A Potential Mechanism for the Formation of Innate Antigens by Antioxidants
Source: PLoS One. 2016 Apr 5;11(4):e0153002. doi: 10.1371/journal.pone.0153002 (PMC4821561; doi:10.1371/journal.pone.0153002)
Supplement: S10 Fig — Normal human serum was used as the first antibody, and HRP-conjugated anti-human IgM antibody was used as the second antibody. (PDF) [file pone.0153002.s010.pdf]

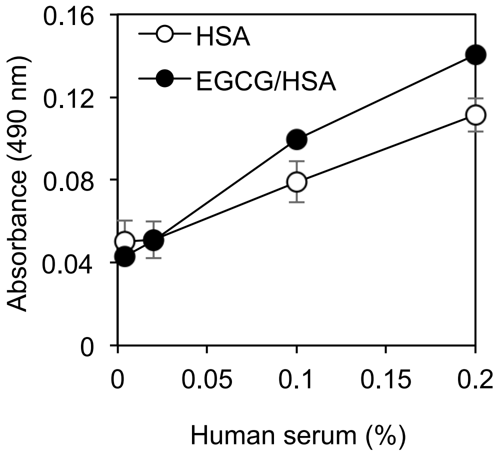

Fig. S10. Cross-reactivity of HSA (*open circle*) and EGCG-treated HSA (*closed circle*) with the IgM of human serum was examined by ELISA. Normal human serum was used as the first antibody, and HRP-conjugated anti-human IgM antibody was used as the second antibody.
