# Supplementary material for: Oxidative Deamination of Serum Albumins by (-)-Epigallocatechin-3-O-Gallate: A Potential Mechanism for the Formation of Innate Antigens by Antioxidants
Source: PLoS One. 2016 Apr 5;11(4):e0153002. doi: 10.1371/journal.pone.0153002 (PMC4821561; doi:10.1371/journal.pone.0153002)
Supplement: S1 Table — (PDF) [file pone.0153002.s011.pdf]

Table S1. Peptides identified by nano-LC-ESI-MS/MS from the EGCG-treated HSA.

Start	End	Observed	Mr (expt)	Mr (calc)	Delta (Da)	Missed clevage	Score	Peptide
191	197	867.3614	866.3541	866.4246	-0.0705	1	26	K.ASSAKQR.L + Lys>ABA-AAS (K)
198	205	1010.4346	1009.4273	1009.5266	-0.0993	1	23	R.LKCASLQK.F + Lys>ABA-AAS (K)
429	436	922.4003	921.3931	921.492	-0.0989	1	44	R.NLGKVGSK.C + Lys>ABA-AAS (K)
440	445	857.3583	856.351	856.4191	-0.0681	1	34	K.HPEAKR.M + Lys>ABA-AAS (K)
539	545	937.4102	936.4029	936.4916	-0.0887	1	41	K.ATKEQLK.A + Lys>ABA-AAS (K)
