# Supplementary material for: Oxidative Deamination of Serum Albumins by (-)-Epigallocatechin-3-O-Gallate: A Potential Mechanism for the Formation of Innate Antigens by Antioxidants
Source: PLoS One. 2016 Apr 5;11(4):e0153002. doi: 10.1371/journal.pone.0153002 (PMC4821561; doi:10.1371/journal.pone.0153002)
Supplement: S9 Fig — (PDF) [file pone.0153002.s009.pdf]

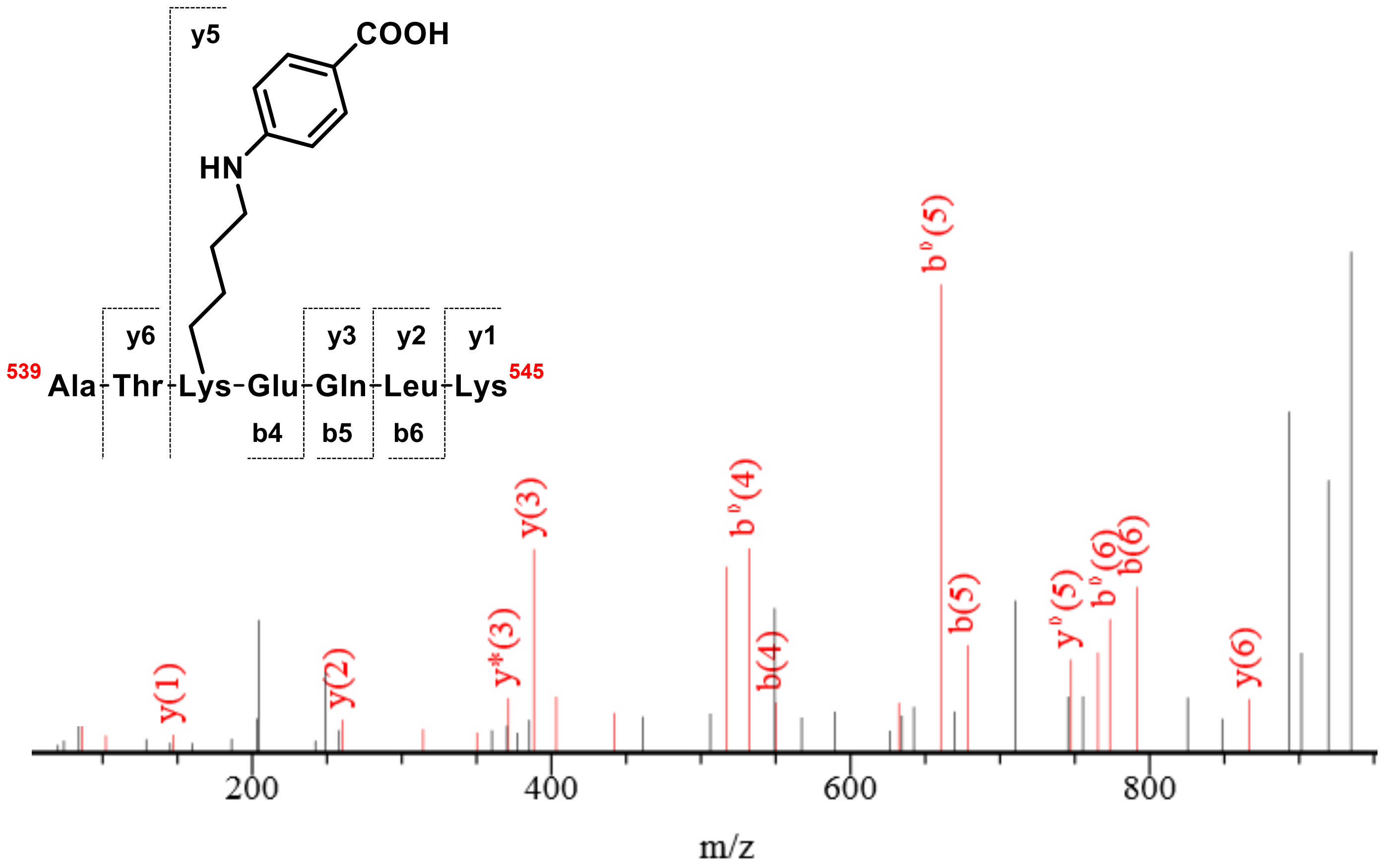

Fig. S9. Nanoelectrospray MS/MS spectrum of the ABA-AAS-containing HSA peptide.
